# Tex264 Binding to SNX27 Regulates Itg*α*5 Receptor Membrane Recycling and Affects Cell Migration

**DOI:** 10.1155/2022/4304419

**Published:** 2022-07-04

**Authors:** Xiao-hui Xu, Qian-wen Yang, Chang-ling Yue, Yun-yi Zhu, Zhao-huan Zhang

**Affiliations:** ^1^School of Preclinical Medicine, Wannan Medical College, Wuhu 241002, China; ^2^School of Life Sciences, Shanghai University, Shanghai 200444, China; ^3^Department of Laboratory Medicine, Changzheng Hospital, Naval Medical University, Shanghai 200003, China

## Abstract

Tex264 is an endoplasmic reticulum (ER) membrane protein that was recently demonstrated to act as an ER-phagy receptor under starvation conditions to mediate endoplasmic reticulum autophagy. However, how Tex264 functions in the central nervous system (CNS) and tumors is unclear. Here, we identified 89 proteins from the rat brain that may specifically interact with Tex264 and confirmed the interaction between sorting nexin 27 (SNX27) and Tex264 by coimmunoprecipitation and immunofluorescence. Our results indicated that Tex264 may promote recycling of membrane proteins from endosomes to the cell plasma membrane by recruiting SNX27 retromer vesicles. siRNA-mediated knockdown of TEX264 in HeLa cells did not affect cell proliferation but did significantly inhibit cell migration through a mechanism that may involve a reduction in SNX27-mediated Itg*α*5 receptor membrane recycling. Results of this study helped identify potential binding Tex264 partners and provide insights into Tex264 functions in the CNS and in tumors.

## 1. Introduction

The endoplasmic reticulum (ER) is a fundamental organelle that plays multiple roles in cell function including glucose homeostasis, lipid biosynthesis, Ca^2+^ stores, protein export, and regulation of cell organelle activity [1]. Proteomic quantification of cellular differences in response to nutritional stress showed that Tex264 binds to ATG8 and then fuses with lysosomes to promote endoplasmic reticulum autophagy, demonstrating that Tex264 acts as an ER-phagy receptor under starvation conditions [2]. The TEX264 protein contains an N-terminal hydrophobic region, cytosolic gyrase inhibitor-like domain, and a C-terminal unstructured intrinsically disordered region [2, 3]. The Tex264 N-terminal transmembrane segment drives its localization to the ER [2]. Meanwhile, the C-terminal region and central GyrI-like domain are exposed in the cytoplasm [2]. Another report indicated that a subset of Tex264 anchors in the nuclear inner membrane and associates with DNA replication forks to counteract TOP1 cleavage complex (TOP1cc) during DNA replication [4]. There is limited information concerning the role of TEX264 in the central nervous system (CNS) and in tumors, although there is evidence that TEX264 does have an ER autophagy-promoting function in these tissues. For example, in Alzheimer-associated neuroinflammation, an anti-inflammatory anthranilate analogue enhances autophagy through mTOR and promotes TEX264-mediated ER turnover [5]. The autophagic cell death- (ACD-) triggering compound loperamide induced reticulophagy and cell death that are predominantly mediated through the reticulophagy receptor RETREG1/FAM134B and TEX264, which together promote proliferation of glioblastoma cells ACD [6].

Together, these previous studies highlight the role of Tex264 in pathological conditions. Furthermore, bioinformatics analyses of Tex264 gene expression profiles based on GEO datasets suggest that Tex264 may be involved in development of oligodendrocyte progenitor cells (OPCs) and neurons as well as in disorders associated with autism. Thus, Tex264 may have a broader function than has been previously reported.

To reveal new biological functions for Tex264, in this study, we explored the Tex264 interactome using global proteomic analyses of rat brain tissues to identify proteins that interact with Tex264. We identified 89 specific binding partners and confirmed the interaction of sorting nexin 27 (SNX27) with Tex264 by coimmunoprecipitation and immunofluorescence. We further found that TEX264 can affect cell migration by regulating SNX27-mediated recycling of Itg*α*5 membrane receptors.

## 2. Materials and Methods

### 2.1. Animals

The animals used in this study were purchased from Shanghai Jiesijie Laboratory Animal Co., Ltd. Animal experiments were conducted according to National Institutes of Health *Guide for the Care and Use of Laboratory Animals*, and the study protocol was approved by the Animal Care and Use Committee of Shanghai University.

### 2.2. Rat Brain Lysis

Rat brain tissues were subjected to two freeze-thaw cycles in lysis buffer (2.5 mM EGTA, 20 mM HEPES, 0.1 M KCl, 1 mM EDTA, 1× protease inhibitor mixture, 0.5% *v*/*v* NP-40, and 1 mM DTT, final pH 7.4) before centrifugation for 1 h at 4°C at 11,000 rpm. The supernatants were transferred to a new tube that was centrifuged again at 11,000 rpm for 1 h at 4°C. The supernatant was transferred to a new tube and labeled as the soluble fraction.

### 2.3. Antibodies and siRNAs

Mouse anti-SNX27 (sc-51570, Santa Cruz), rabbit anti-Tex264 (ab272575, Abcam), rabbit anti-Itg*α*5 (A0832, ABclonal), and HRP-conjugated monoclonal anti-GAPDH (KC-5G5, Kangcheng) were used. For Tex264 knockdown, siRNA (SiTEX264) had sequences that corresponded to previous reports [3] and were as follows: antisense 5′-UGUCAUAGUAGACAGCGAUGGAGCG-3′ and sense 5′-CGCUCCAUCGCUGUCUACUAUGACA-3′. The control scramble siRNA had nucleotide changes at 5 random positions.

### 2.4. GST Protein Expression and Purification

Plasmids were constructed by Generay Biotech Co., Ltd. (Shanghai). GST-Tex264 fusion proteins were expressed in *Escherichia coli* BL21 (DE3) cells. Expression of fusion proteins was induced with 0.2 mM IPTG at 25°C for 6 h. The cells were centrifuged in 2 × 250 mL volumes at 4,000 g for 10 min at 4°C. The bacterial pellets were resuspended in 25 mL lysis buffer (PBS, 1 mM PMSF, 1 mg/mL lysozyme, pH 7.4) and incubated for 30 min before addition of 0.5% *v*/*v* Triton X-100 with 1 mM PMSF and 5 mM DTT incubation on ice for another 30 min. The lysates were then centrifuged at 12,000g for 30 min at 4°C. The pellets were discarded, and the supernatants were incubated with 1 mL of a 50% *v*/*v* slurry of glutathione-Sepharose beads for 1 h at 4°C. The mixtures were centrifuged at 3,000 rpm for 1 min at 4°C, and the resulting pellets were washed once with PBS (10 mL, pH 7.4) followed by centrifugation at 3,000 rpm for 1 min at 4°C. The glutathione-Sepharose beads were then washed 3 times in wash buffer (1 mL, 100 mM KCl, 1 mM DTT, 20 mM HEPES, and 1× protease inhibitor cocktail, pH 7.4) at room temperature and then with binding buffer (2.5 mM EGTA, 1 mM EDTA, 20 mM HEPES, 1 mM DTT, 0.1 M KCl, and 1× protease inhibitor cocktail, pH 7.4). A Lowry assay was used to determine the concentration of the purified protein.

### 2.5. GST Pull-Down Assay

To exclude proteins with nonspecific binding, the soluble fraction of rat brain lysates was preincubated with beads followed by GST-beads. The remaining supernatants were incubated with GST-Tex264-beads at 4°C for 2 h before washing with binding buffer (1% NP40, 1× protease inhibitors, and 2 mM DTT in 1× PBS) and washing buffer (20 mM HEPES, 2.5 mM EGTA, 1× protease inhibitor cocktail, 0.1 M KCl, 1 mM EDTA, and1 mM DTT with 25 mM GSH, pH 7.4). Finally, the proteins were concentrated in PBS using an ultrafiltration column (Amicon® Ultra filters) with a 3 kDa cutoff.

### 2.6. Mass Spectrometry and Data Analysis

The purified proteins were sent to the Mass Spectrometry Core at Shanghai University for LC-MS/MS analysis, which was performed on a Q Exactive mass spectrometer and coupled with Easy-nLC (Thermo Fisher Scientific) running in positive ion mode. A data-dependent top 10 method was used to acquire MS data that allowed dynamic selection of the most abundant precursor ions for HCD fragmentation from the survey scan (300-1800 *m*/*z*). Automatic gain control (AGC) of the target (3e6), a 10 ms maximum inject time, and 40 s dynamic exclusion duration were used. Survey scans were acquired at a resolution of 70,000 at *m*/*z* with a 2 *m*/*z* 200 isolation width, and the HCD spectral resolution was 17,500 at *m*/*z* 200. The normalized collision energy was 30 eV with a 0.1% under fill ratio. Mascot2.2 software was used to analyze the MS data with the following parameters: trypsin enzyme, rat (35719) taxonomy, two missed cleavage sites, UniProt database, 20 ppm MS/MS tolerance, carbamidomethylation of cysteine and oxidation of methionine as fixed and dynamic modifications, protein FDR 0.01, peptide FDR 0.01, and filter by score of 20.

### 2.7. Immunofluorescence Staining

Brain slices from Sprague-Dawley rats or cultured cells were washed in 0.01 M PBS and then fixed with 4% paraformaldehyde (PFA) for 30 min at room temperature (RT). The samples were treated with 0.1% Triton X-100 in 0.01 M PBS for 30 min, blocked with 1% bovine serum albumin (BSA) in 0.01 M PBS (RT, 1 h), and subsequently incubated with a primary antibody at 4°C overnight. After washing 5 times, the samples were incubated with fluorescently labeled secondary antibody (1 : 100, Santa Cruz).

### 2.8. 5-Ethynyl-2′-deoxyuridine (EdU) Labeling

HeLa cells seeded in 24-well plates at 1.5 × 10^5^ cells/mL were cultured in EdU solution for 1 d and then fixed for 0.5 h at RT in 4% PFA. The cells were then permeabilized with 0.5% Triton X-100 in PBS for 20 min, incubated with 1× Apollo® reaction cocktail for 30 min, stained with DAPI for 15 min, and imaged under a fluorescent microscope. The proliferation rate was defined as the ratio of EdU-positive cells (red) to DAPI-positive cells (blue).

### 2.9. Surface Labeling of Itg*α*5

Surface labeling of membrane proteins was carried out as previously described [7]. Briefly, HeLa cells were transfected with siTex264 or scramble siRNA and 72 h later were seeded on coverslips before incubation with a mouse anti-Itg*α*5 antibody (1 : 100, 1% BSA and 5% FBS in DMEM) for 60 min at 37°C. The cells were then washed four times with culture medium and treated with 4% PFA for 30 min at RT. Then, the cells were washed three times and incubated with secondary antibodies (Alexa 488 donkey anti-rabbit IgGs) for 2 h. Microscopic images were acquired using an Axio Observer A1 inverted confocal microscope.

### 2.10. Wound Healing Assay

HeLa cells were transfected with siTex264 or scramble siRNA, seeded on 6-well plates, and cultured until confluency was achieved. A 200 *μ*L pipette tip was used to make a straight scratch across the cell layer to form a wound. After removing the medium, cells were washed 2 times with 0.01 M PBS, and then, 1.5 mL DMEM containing 2% FBS was added. The scratched area was photographed 0 h, 6 h, 12 h, and 24 h after wounding. Images were analyzed using ImageJ (NIH). The cell migration was quantified and expressed as the average width (pixels) of the scratch area.

### 2.11. Statistics

GraphPad Prism 5 software was used for statistical analysis of all data, and Student's *t*-test or one-way analysis of variance methods was used as indicated.

## 3. Results

### 3.1. Cloning and Expression of Rat Tex264 in *E. coli*

**Figure 1:**
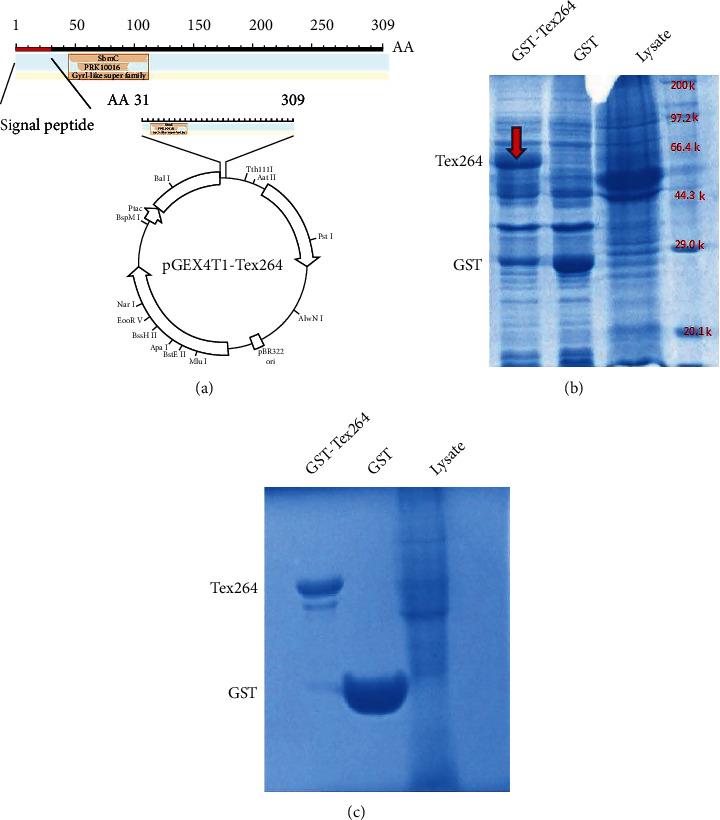
Construction of the GST-TEX264 expression vector and GST-TEX264 fusion protein expression. (a) Construction of the TEX264 expression plasmid using the pGEX4T1 vector. (b) Coomassie blue staining to confirm successful expression of GST-TEX264 and GST protein. (c) Purified GST-TEX264 and GST protein assessed by Coomassie blue staining.

In this study, Tex264 from the Rattus norvegicus testis NM_001007665.1 (accession number) was cloned into the pGEX-4T-1 expression vector (Figure 1(a)). The construct did not include the signal peptide, such that the expressed Tex264 had 840 nucleotides encoding 279 amino acids.

To confirm successful expression of the Tex264 GST fusion protein, cells were transformed with pGEX-4T1-Tex264 (GST-Tex264) or empty pGEX-4T1 vector (control). Total cell lysates of the transfected cells were analyzed by SDS-PAGE with Coomassie blue staining, which showed a major band for GST-Tex264 that corresponded to the expected molecular weight of around 60 kDa (Figure 1(b)). After purification, both GST-Tex264 and GST yielded clear bands at the expected positions on PAGE gels (Figure 1(c)).

### 3.2. LC-MS/MS Analysis to Identify Proteins Interacting with Tex264

To purify Tex264-specific binding proteins, we used a preclearing strategy similar to that described in our previous work [7–9]. After clearing and pull-down, rat brain proteins were analyzed by LC/LC-MS/MS (tandem liquid chromatography-tandem mass spectrometry). Raw data were analyzed with Mascot2.2 software, using a high stringency filtering (score ≥ 20), and then compared to the UniProt database (rat (35719)). Among the 124 proteomes, 260 interacting proteins were identified (Supplemental Figure 1 and Supplemental Table 1). Comparison with GST pull-down proteins removed several common nonspecific binding proteins such as microtubule and microfilament protein and myosin, leaving 89 possible Tex264-specific binding proteins. Based on information in the BioGRID database, we exported 57 validated TEX264-interacting proteins, which were compared with the 89 proteins that we identified in this study and displayed as a Venn diagram (http://bioinformatics.psb.ugent.be/webtools/Venn/). Of the 89 proteins, 4 (GABARAP, F13A1, ASAP2, and CSNK1A1) had been previously described. The remaining 85 proteins were novel Tex264-interacting partners.

### 3.3. KEGG and GO Analysis of Tex264-Interacting Proteins

Function annotation by KEGG and GO analyses using the online software Metascape and PANTHER15.0 was next carried out. The GO analyses showed the most relevant and meaningful enriched items (*p* < 0.01) among the TEX264 binding proteins in terms of biological function (Supplemental Figure 2). The 89 TEX264 binding proteins were classified and annotated according to cellular compartment (CC) (Supplemental Figure 3), biological process (BP) (Supplemental Figure 3), protein class (PC), and molecular function (MF) (Supplemental Figure 4). TEX264-interacting proteins were involved in a range of BP including development, cell adhesion, growth, signaling pathway, and metabolic process. CC analysis revealed 18 membrane proteins, 27 organellar proteins, and 2 proteins in the synapse. MF analysis revealed that 29.06% were involved in binding, 27.9% have catalytic activity, 10.46% have transcription factor regulation activity, and 3.48% have transporter activity. PC analysis revealed 6.7% as metabolite interconversion enzymes, 5.6% are gene-specific transcriptional regulators, 2.2% are membrane trafficking proteins, 4.5% are nucleic acid binding proteins, 4.5% are cytoskeleton proteins, and 3.4% are transporters.

### 3.4. SNX27 Interacts with TEX264

Based on a search of MS data that revealed more frequent appearance of SNX27 peptides, GST-TEX264 pull-down proteins were immunoblotted with an anti-SNX27 antibody. This immunoblotting produced a specific band in brain lysates or in GST-TEX264-transfected cells but not in the GST (control) group. This result suggested that bacterially expressed TEX264 could specifically bind to rat brain SNX27 in vitro.

**Figure 2:**
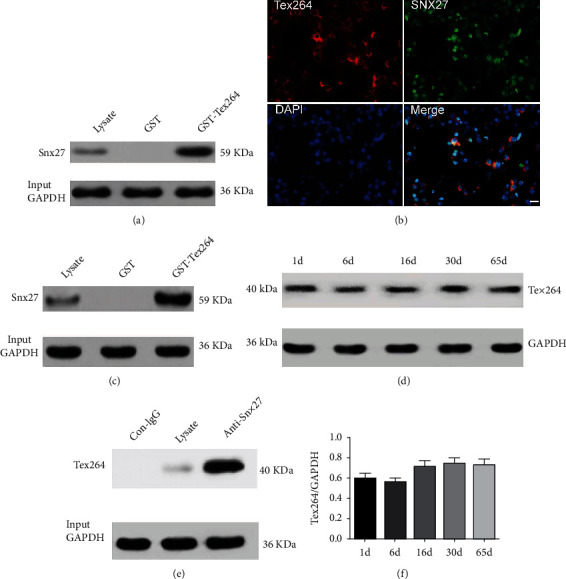
Identification of SNX27 as a TEX264-binding protein. (a) GST pull-down assay to confirm direct binding of GST-TEX264 with SNX27 in vitro. Rat brain lysates were incubated with GST-TEX264 or GST-coated beads. After washing and elution, western blotting for SNX27 on brain lysates was carried out with an input control (input) performed in parallel. (b, c) Immunoprecipitation (IP) shows interaction of endogenous TEX264 (b) and SNX27 (c) in rat brain tissues. Equal amounts of total lysates were set as the input control. Control IgG was used as the immunoprecipitation control. (d) Immunofluorescence staining to show colocalization of TEX264 with SNX27 in P0 rat brain cortex sections. Blue: DAPI staining of nuclei; green: anti-SNX27 staining; red: anti-TEX264 antibody staining. Bar = 20 *μ*m. (e) Western blotting to detect TEX264 expression in rat brains at different developmental stages. (f) Quantification of TEX264 expression. TEX264 blots were compared with GAPDH levels, and a ratio was determined and then presented as ratio data. Data shown are the mean ± SD of three independent experiments. No significant difference: one-way ANOVA statistics.

**Figure 3:**
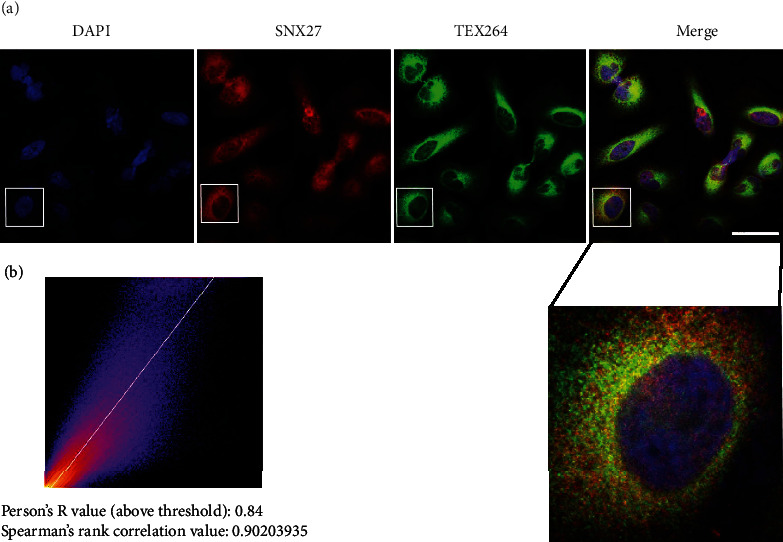
TEX264 colocalized with SNX27 in HeLa cells. (a) Immunofluorescence staining shows TEX264 colocalization with SNX27 in HeLa cells. Blue: DAPI staining of nuclei; green: anti-SNX27 staining; red: anti-TEX264 antibody staining. Bar = 20 *μ*m. (b) Immunofluorescence images were analyzed for TEX264 and SNX27 colocalization using the Coloc2 ImageJ plug-in. The 2D intensity histogram shows that the majority of these proteins colocalized, and the colocalization coefficients were higher.

We subsequently performed coimmunoprecipitation and immunoblotting experiments to further confirm whether endogenous TEX264 and SNX27 can form natural complexes in the rat brain. An anti-TEX264 antibody or control IgG (control) was first used to immunoprecipitate rat brain lysates with total brain lysates used in parallel as an input control. Immunoblotting was then performed with an anti-SNX27 antibody. The results showed that SNX27 was detected in immunoprecipitates of the anti-TEX264 antibody group and brain lysate group (input control), whereas no SNX27 was detected in immunoprecipitates of the control IgG group (Figure 2(b)). This result demonstrates that endogenous TEX264 can form a coprecipitable protein complex with endogenous SNX27 in the rat brain. Rat brain lysates were also immunoprecipitated with the anti-SNX27 antibody and then immunoblotted against TEX264, which was detected only in anti-SNX27 immunoprecipitates and brain lysates. Immunofluorescence imaging also showed that endogenous TEX264 and SNX27 are widely expressed in rat brain tissue and partially colocalize in neuronal cells (Figure 2(d)). High-power confocal fluorescence images showed that TEX264 and SNX27 are highly colocalized in cultured HeLa cells (Figure 3).

### 3.5. TEX264 Knockdown Affects Cell Migration

**Figure 4:**
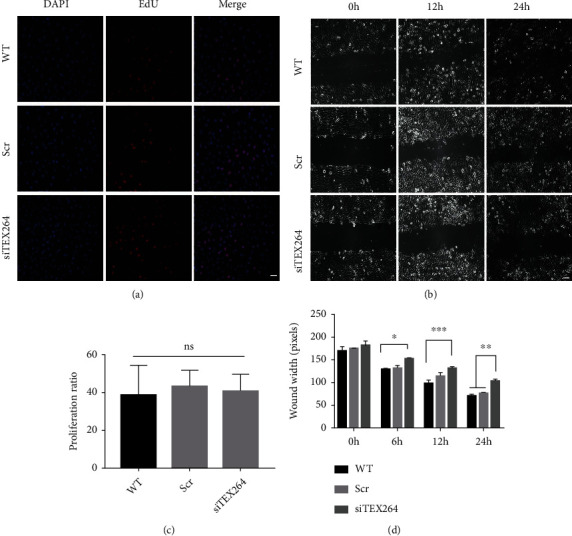
TEX264 knockdown affects HeLa cell migration but not proliferation. (a) HeLa cells were transfected with siTEX264 or scramble siRNA (Scramble) and seeded on 24-well plates for the EdU assay. The proliferation rate was defined as the ratio of EdU-positive cells (red) to DAPI-positive cells (blue). (b) Quantitative data for the EdU assay. Data are the mean ± SD (*n* = 3); nd = no difference. (c) Migration of TEX264-knockdown HeLa cells determined using a wound-healing assay. HeLa cells were transfected with siTEX264 or scramble siRNA (Scramble), seeded on 6-well plates, and cultured to confluency. WT (untransfected HeLa cells). Cell-free gaps were created, and images of the closed gaps were taken at the indicated time point. The wound width was measured at five sites. (d) TEX264 knockdown significantly inhibits HeLa cell migration. ^∗^*p* < 0.01, ^∗∗^*p* < 0.005, and ^∗∗∗^*p* < 0.001 compared to the Scramble or WT cells.

To further understand the physiological role of TEX264 in cells, we knocked down TEX264 expression in HeLa cells with TEX264-specific siRNA. Proliferation of HeLa cells with TEX264 knockdown was not changed, but substantial changes in cell migration were seen. No difference in positive Edu staining was seen in staining when comparing TEX264 knockdown and control groups (Figures 4(a) and 4(b)). These results were supported by wound healing experiments showing that TEX264 knockdown significantly inhibited HeLa cell migration (Figures 4(c) and 4(d)).

### 3.6. Knockdown of TEX264 Affects the Cellular Localization of SNX27 and Membrane Localization of Itg*α*5

**Figure 5:**
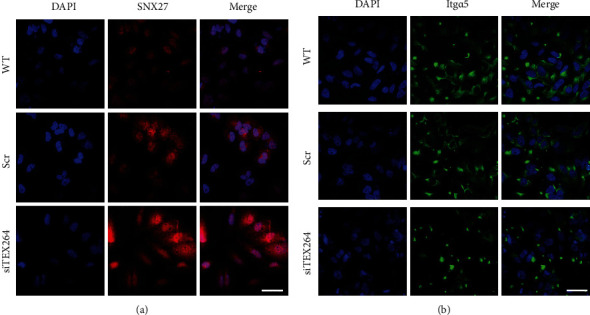
TEX264 knockdown affects SNX27 cell localization and inhibits membrane localization of Itg*α*5 integrin receptors. (a) HeLa cells were transfected with siTEX264 or scramble siRNA and stained with an antibody against SNX27 24 h later. Scale bar, 20 *μ*m. (b) HeLa cells transfected with siTEX264 or scramble siRNA. After 48 h, cells were live-stained with an antibody against Itg*α*5 48 h after transfection to reveal surface levels of the Itg*α*5 integrin receptor. Scale bar, 20 *μ*m.

SNX27 vesicles are reported to be related to recycling of many membrane receptors and membrane proteins. Membrane receptors like integrin receptors are closely related to cell migration. Thus, we speculate that inhibition of TEX264 will impact SNX27-mediated recycling of membrane proteins to the cell membrane that results in aberrant membrane distribution of integrin receptors and in turn decreased cell migration. To examine this possibility, we assessed SNX27 membrane localization by immunofluorescence and cell surface staining to quantify membrane distribution of the Itg*α*5 integrin receptor, which is recycled by SNX27. Cells with TEX264 knockdown had more diffuse distribution of SNX27, and the cell size increased slightly (Figure 5(a)). Staining intensity for membrane surface Itg*α*5 was lower in the TEX264 knockdown group compared to control cells, suggesting that TEX264 plays a role in Itg*α*5 membrane localization (Figure 5(b)).

## 4. Discussion

Although several interacting proteins have been reported for TEX264, the function of TEX264 in the CNS and tumors is largely unclear. The continuous and stable expression of TEX264 in the brain tissue of developing and young adult rats (Figures 2(e) and 2(f)) suggests that TEX264 may play an important role in maintaining normal physiological function of the CNS.

Our mass spectrometry results showed that the most frequent peptides from TEX264 pull-down corresponded to sorting nexin 27 (SNX27) (Supplemental Table 1). This result indicated that SNX27 likely interacts with TEX264 in rat brains, and results of biochemical and immunofluorescence experiments supported this possibility (Figures 2 and 3). SNX27 belongs to the sorting nexin protein family, whose members are involved in endosome recycling as well as endosomal sorting and signaling. SNX27 facilitates recycling of endocytic transmembrane proteins from endosomes to the plasma membrane [10]. Many cell surface proteins such as the copper transporter ATP7A, glucose transporter 1 (GLUT1), and numerous signaling receptors can interact with SNX27 and require SNX27-endosomal sorting to prevent lysosomal degradation and maintain plasma membrane surface levels [11–13].

SNX27 can directly interact with K-ras GTPase and recruit K-ras into SNX27-positive endosomes through a Ca^2+^/CaM-dependent mechanism that promotes synaptic transmission of homomeric GluA1 receptors. Knockdown of SNX27 inhibits LTP and related transport of AMPAR to the membrane. Snx27-knockout mice have severe neuronal defects in the hippocampus and cortex [14, 15].

TEX264 expression is increased in tumor cells [6, 16]. To further understand the physiological functions of TEX264 in culture cells, we used siTEX264 to knock down endogenous TEX264 expression in HeLa cells. Cells with TEX264 knockdown had significantly inhibited migration that could be related to cell surface expression of several relevant receptors. Integrin receptors are known to be closely related to cell migration [17], and SNX27 is reported to be closely related to the membrane display of integrin receptor subtype Itg*α*5 [18]. SNX27 knockdown indeed reduces Itg*α*5 membrane localization. The interaction between TEX264 and SNX27 we found here together with the role for SNX27 in regulating membrane receptor recycling suggests that the decrease in HeLa cell migration may indicate a role for TEX264 in SNX27-mediated membrane insertion of Itg*α*5. The interaction of TEX264 and SNX27 under physiological conditions could promote SNX27-mediated recycling of some membrane proteins and membrane receptors to the cell membrane that are important for maintaining normal cell physiological functions. Given the effect of the TEX264 and SNX27 interaction on tumor cell migration, development of drugs that block this interaction could inhibit tumor invasion and metastasis in vivo. Furthermore, SNX27 and Itg*α*5 are expressed in the CNS, suggesting that TEX264 could also promote migration of neural precursor cells during early CNS development.

In summary, we identified 85 new TEX264 binding proteins using GST-pulldown and LS-MS technology. These proteins are ubiquitous throughout cells and have many functions, including endosome recycling, mitochondrial organization, transcription factor binding, RNA splicing, and apoptosis. The discovery of these new TEX264 binding proteins indicates that TEX264 not only promotes ER autophagy but also plays a role in a broad range of biological functions. To better understand the function of TEX264, future studies to validate the physiological relevance of TEX264-interacting proteins are needed. Taken together, the results of the current studies can form the basis for future research on the role of TEX264 in physiological processes and in disease pathology.

## Data Availability

The data used to support the findings of this study are available from the corresponding author upon request.

## Supplementary Materials

Supplementary 1 Supplementary Figure 1: Venn diagram depicting Tex264 binding proteins. Venn diagram showing unique and shared proteins between 2 groups (1 is that 57 proteins had been reported binding with Tex264, and 2 is our newly identified 85 proteins). The protein details are listed in the lower table.

Supplementary 2 Supplementary Figure 2: GO analysis of Tex264-interacting proteins. (A) GO classification of the Tex264 interactors. (B) Meanwhile, the enriched pathways were further listed in the lower table.

Supplementary 3 Supplementary Figure 3: classification of Tex264-interacting proteins based on the biological process (BP) and cellular compartment (CC). Pie chart representation of Gene Ontology classification of putative molecular functions of Tex264-interacting proteins from where they localized (CC) and the biological processes (BP) in which they are involved.

Supplementary 4 Supplementary Figure 4: classification of Tex264-interacting proteins based on protein class (PC) and molecular function (MF). Pie chart representation of Gene Ontology classification of Tex264-interacting proteins according to protein class (PC) and molecular function (MF).

Supplementary 5 Supplementary Figure 5: the PPI map of Tex264-interacting proteins. The PPI (protein-protein interaction) map based on GST-Tex264-specific pull-down proteins analyzed by the STRING database.

Supplementary 6 Supplementary Table 1: mass spectrometry results of Tex264-specific pull-down proteins.
