# Supplementary material for: Tex264 Binding to SNX27 Regulates Itgα5 Receptor Membrane Recycling and Affects Cell Migration
Source: Biomed Res Int. 2022 Jul 4;2022:4304419. doi: 10.1155/2022/4304419 (PMC9274233; doi:10.1155/2022/4304419)
Supplement: Supplementary 1 — Supplementary Figure 1: Venn diagram depicting Tex264 binding proteins. Venn diagram showing unique and shared proteins between 2 groups (1 is that 57 proteins had been reported binding with Tex264, and 2 is our newly identified 85 proteins). The protein details are listed in the lower table. [file 4304419.f1.pdf]

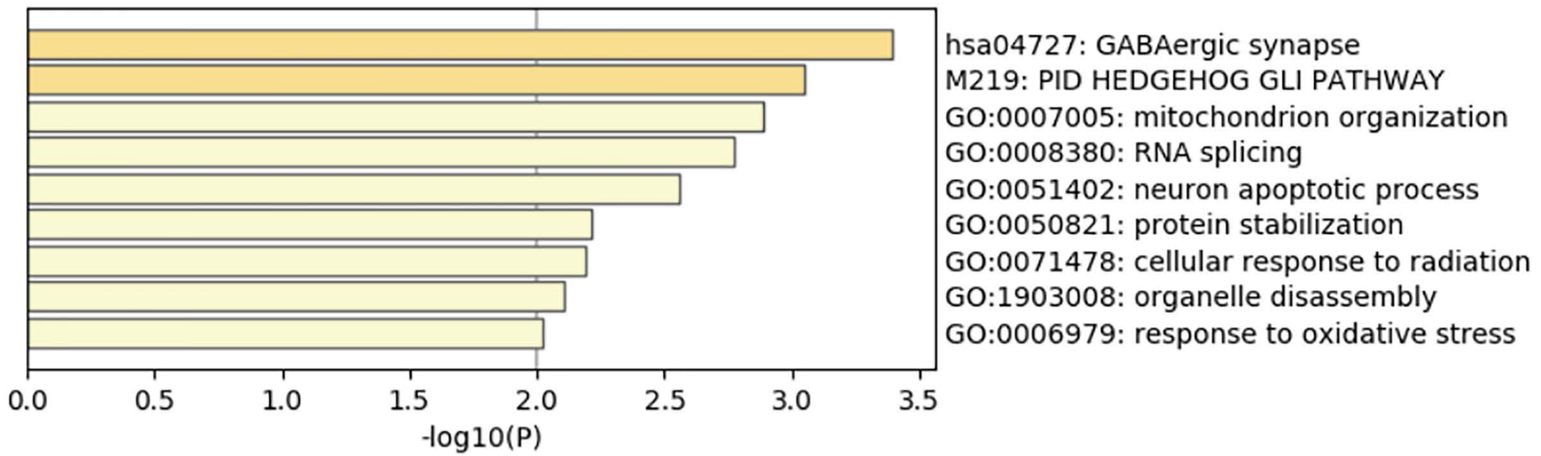

GO	Category	Description	Count	%	Log10(P)	Log10(q)
hsa04727	KEGG Pathway	GABAergic synapse	4	4.21	-3.39	0.00
M219	Canonical Pathways	PID HEDGEHOG GLI PATHWAY	3	3.16	-3.05	0.00
GO:0007005	GO Biological Processes	mitochondrion organization	8	8.42	-2.89	0.00
GO:0008380	GO Biological Processes	RNA splicing	7	7.37	-2.77	0.00
GO:0051402	GO Biological Processes	neuron apoptotic process	5	5.26	-2.56	0.00
GO:0050821	GO Biological Processes	protein stabilization	4	4.21	-2.21	0.00
GO:0071478	GO Biological Processes	cellular response to radiation	4	4.21	-2.19	0.00
GO:1903008	GO Biological Processes	organelle disassembly	3	3.16	-2.11	0.00
GO:0006979	GO Biological Processes	response to oxidative stress	6	6.32	-2.02	0.00
