# Supplementary figures and images for: Tex264 Binding to SNX27 Regulates Itgα5 Receptor Membrane Recycling and Affects Cell Migration
Source: Biomed Res Int. 2022 Jul 4;2022:4304419. doi: 10.1155/2022/4304419 (PMC9274233; doi:10.1155/2022/4304419)

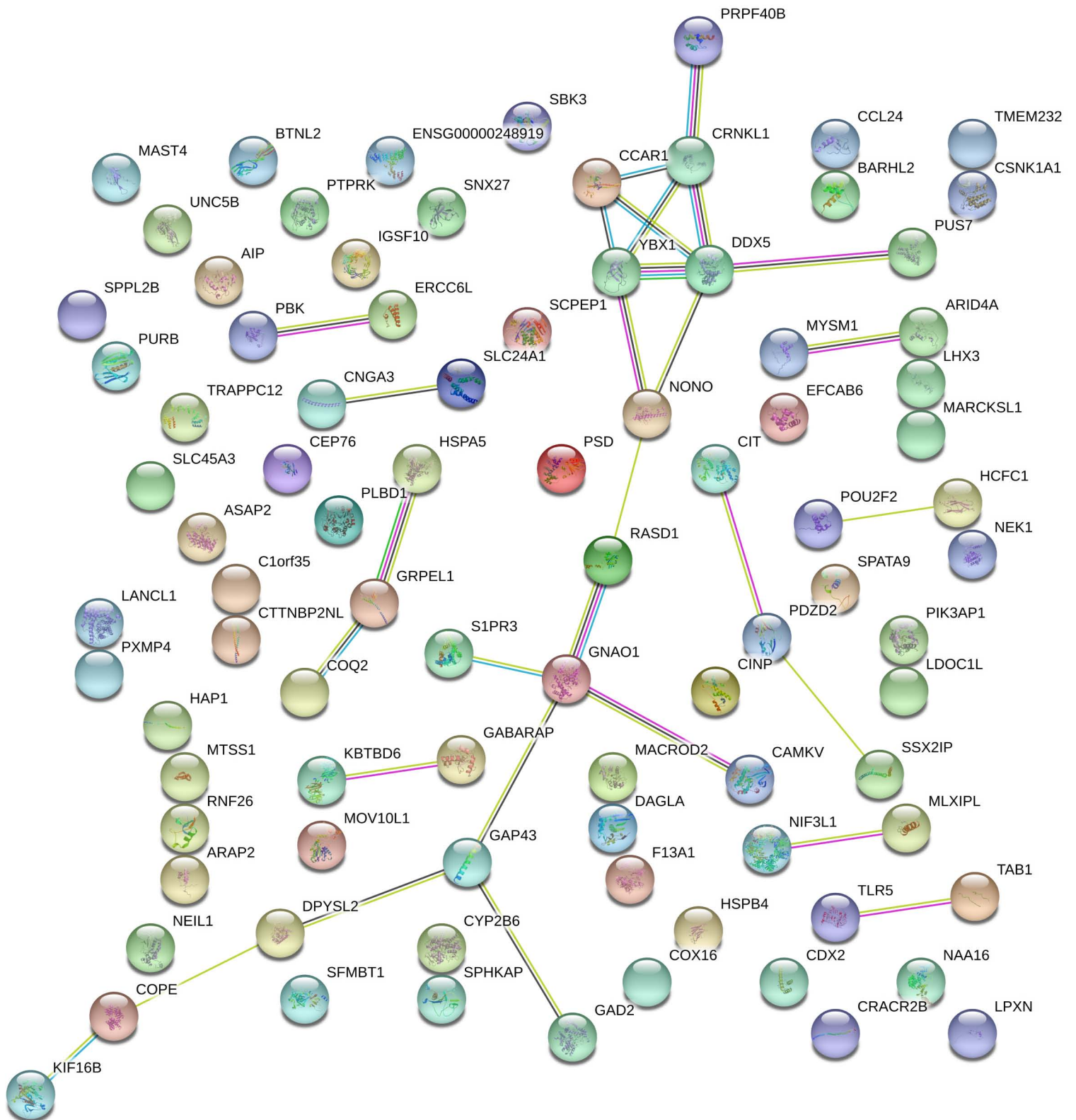

Supplement: Supplementary 2 — Supplementary Figure 2: GO analysis of Tex264-interacting proteins. (A) GO classification of the Tex264 interactors. (B) Meanwhile, the enriched pathways were further listed in the lower table. [file 4304419.f2.pdf]
