# Supplementary material for: Tex264 Binding to SNX27 Regulates Itgα5 Receptor Membrane Recycling and Affects Cell Migration
Source: Biomed Res Int. 2022 Jul 4;2022:4304419. doi: 10.1155/2022/4304419 (PMC9274233; doi:10.1155/2022/4304419)
Supplement: Supplementary 3 — Supplementary Figure 3: classification of Tex264-interacting proteins based on the biological process (BP) and cellular compartment (CC). Pie chart representation of Gene Ontology classification of putative molecular functions of Tex264-interacting proteins from where they localized (CC) and the biological processes (BP) in which they are involved. [file 4304419.f3.pdf]

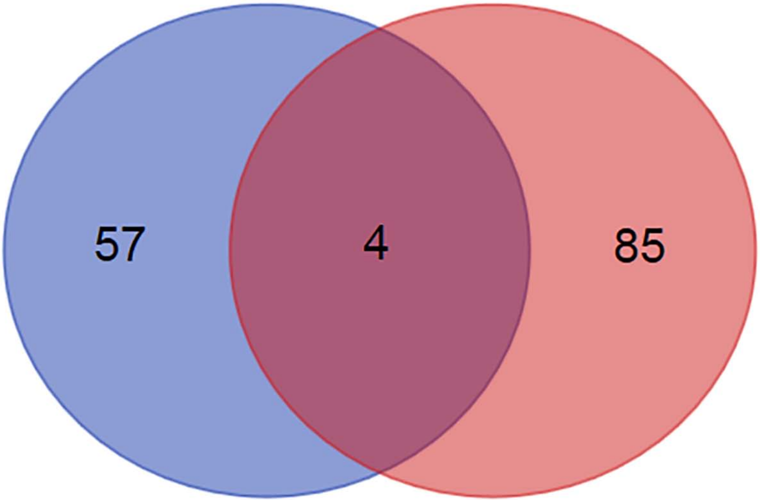


| Names | total | elements |
|---|---|---|
| user_list1 user_list2 | 4 | ASAP2 GABARAP F13A1 CSNK1A1 |
| user_list1 | 57 | TMEM216 EDA JPH1 CEP131 HFE ACTBL2 MAP4K4 CSNK1D ENTPD7 PDZD8 ABCC10 HMOX1 SPRTN POTEI VANGL2 APEX1 UPK3BL TMEM79 OSBPL8 PLD2 MTCH2 CSNK1E TMEM30A EVA1C SNX14 TCTN2 TYW1B DIP2A FAM105A SPINT2 PLD1 MAPK6 TERF2 CTDNEP1 CHEK2 PLEKHA4 ISLR CA9 STAT1 SUN1 SEMA4C LC3 GDPD5 PER1 MCM10 PNPLA6 FOXK1 C1orf43 RHBDD1 RHBDF2 SYVN1 MINK1 PDE3B NKX3-1 TOP1 RNF4 PLEKHG4 |
| user_list2 | 85 | YBX1 BARHL2 SPATA9 SFMBT1 CCAR1 PTPRK HSPA5 PUS7 ATP5MF-PTCD1 GAP43 BTNL2 COX16 CRNKL1 CINP KBTBD6 CEP76 DDX5 PURB PRPF40B COA8 ARAP2 None RTL6 MAST4 MACROD2 SLC45A3 CIT MTSS1 MARCKSL1 DAGLA NIF3L1 DPYSL2 UNC5B CTTNBP2NL PIK3AP1 PLBD1 TRAPPC12 KIF16B HAP1 CAMKV NONO SPPL2B CRYAA AIP SCPEP1 IGSF10 PBK CDX2 POU2F2 PXMP4 GRPEL1 RASD1 SNX27 CRACR2B LPXN COQ2 RNF26 PDZD2 NEK1 MOV10L1 CYP2B6 ERCC6L PSD SLC24A1 MYSM1 SSX2IP COPE NEIL1 MLXIPL HCFC1 LANCL1 TMEM232 TLR5 SPHKAP CNGA3 EFCAB6 LHX3 GAD2 NAA16 SBK3 S1PR3 C1orf35 TAB1 ARID4A GNAO1 |
