# Supplementary material for: Tex264 Binding to SNX27 Regulates Itgα5 Receptor Membrane Recycling and Affects Cell Migration
Source: Biomed Res Int. 2022 Jul 4;2022:4304419. doi: 10.1155/2022/4304419 (PMC9274233; doi:10.1155/2022/4304419)
Supplement: Supplementary 4 — Supplementary Figure 4: classification of Tex264-interacting proteins based on protein class (PC) and molecular function (MF). Pie chart representation of Gene Ontology classification of Tex264-interacting proteins according to protein class (PC) and molecular function (MF). [file 4304419.f4.pdf]

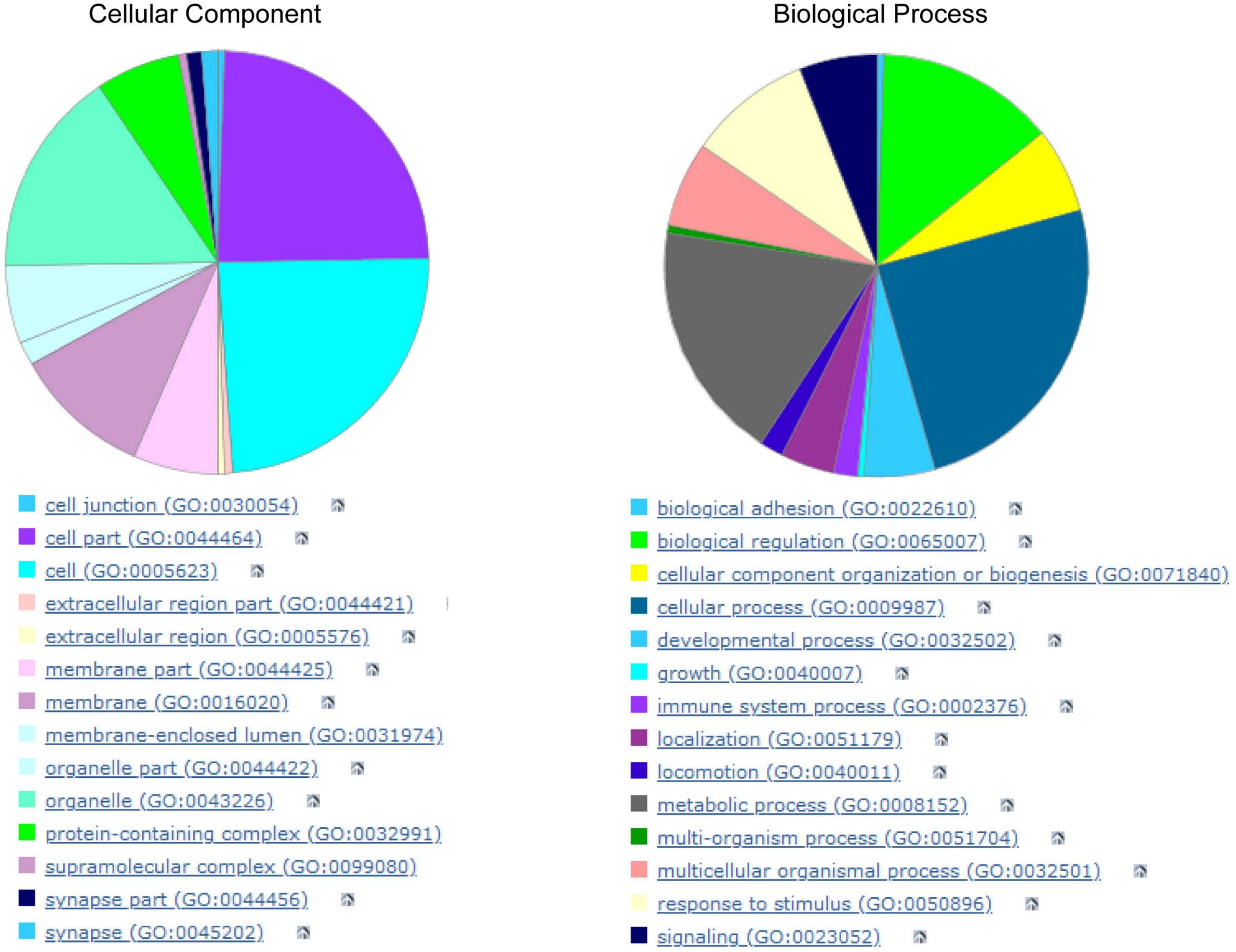
Cellular Component
cell junction (GO:0030054)
cell part (GO:0044464)
cell (GO:0005623)
extracellular region part (GO:0044421)
extracellular region (GO:0005576)
membrane part (GO:0044425)
membrane (GO:0016020)
membrane-enclosed lumen (GO:0031974)
organelle part (GO:0044422)
organelle (GO:0043226)
protein-containing complex (GO:0032991)
supramolecular complex (GO:0099080)
synapse part (GO:0044456)
synapse (GO:0045202)
Biological Process
biological adhesion (GO:0022610)
biological regulation (GO:0065007)
cellular component organization or biogenesis (GO:0071840)
cellular process (GO:0009987)
developmental process (GO:0032502)
growth (GO:0040007)
immune system process (GO:0002376)
localization (GO:0051179)
locomotion (GO:0040011)
metabolic process (GO:0008152)
multi-organism process (GO:0051704)
multicellular organismal process (GO:0032501)
response to stimulus (GO:0050896)
signaling (GO:0023052)
