# Supplementary material for: Tex264 Binding to SNX27 Regulates Itgα5 Receptor Membrane Recycling and Affects Cell Migration
Source: Biomed Res Int. 2022 Jul 4;2022:4304419. doi: 10.1155/2022/4304419 (PMC9274233; doi:10.1155/2022/4304419)
Supplement: Supplementary 5 — Supplementary Figure 5: the PPI map of Tex264-interacting proteins. The PPI (protein-protein interaction) map based on GST-Tex264-specific pull-down proteins analyzed by the STRING database. [file 4304419.f5.pdf]

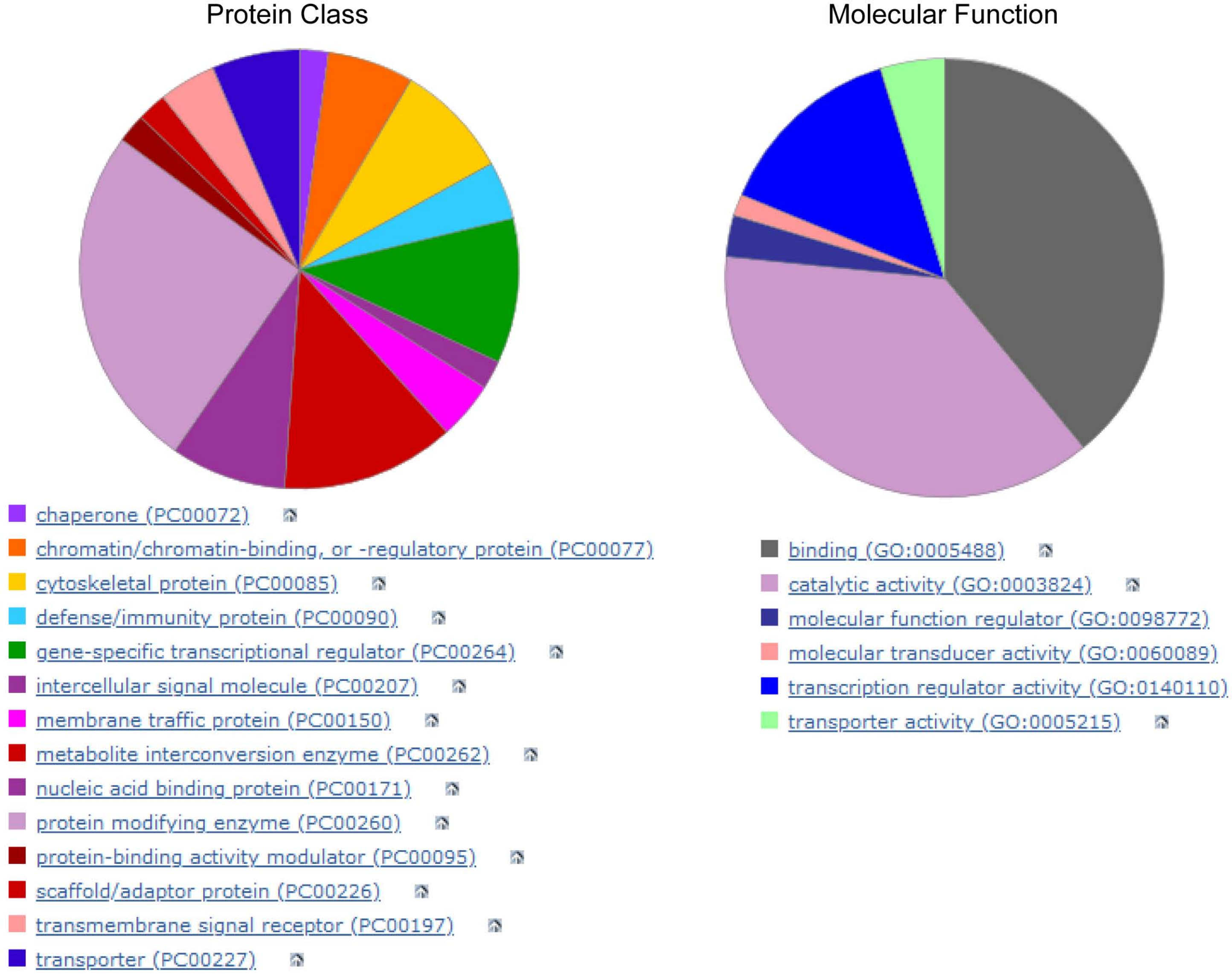
Protein Class
chaperone (PC00072)
chromatin/chromatin-binding, or -regulatory protein (PC00077)
cytoskeletal protein (PC00085)
defense/immunity protein (PC00090)
gene-specific transcriptional regulator (PC00264)
intercellular signal molecule (PC00207)
membrane traffic protein (PC00150)
metabolite interconversion enzyme (PC00262)
nucleic acid binding protein (PC00171)
protein modifying enzyme (PC00260)
protein-binding activity modulator (PC00095)
scaffold/adaptor protein (PC00226)
transmembrane signal receptor (PC00197)
transporter (PC00227)
Molecular Function
binding (GO:0005488)
catalytic activity (GO:0003824)
molecular function regulator (GO:0098772)
molecular transducer activity (GO:0060089)
transcription regulator activity (GO:0140110)
transporter activity (GO:0005215)
