# Supplementary material for: Tex264 Binding to SNX27 Regulates Itgα5 Receptor Membrane Recycling and Affects Cell Migration
Source: Biomed Res Int. 2022 Jul 4;2022:4304419. doi: 10.1155/2022/4304419 (PMC9274233; doi:10.1155/2022/4304419)
Supplement: Supplementary 6 — Supplementary Table 1: mass spectrometry results of Tex264-specific pull-down proteins. [file 4304419.f6.pdf]

	Referenc	PepCount	UniquePe	CoverPerc	MW	PI		
	FileScan	Sequence	MH+	Diff(MH+)	Charge	Rank	Score	DeltaScor
$1-1	tr|Q5XIF	286	18	71.20%	33770.6	4.94		
	R15076_4_	K.CWELVE	3597.625	-0.00086	3	1	33.64	0
	R15076_4_	K.CWELVE	3597.625	-0.00086	3	1	53.24	0
	R15076_4_	K.CWELVE	3597.625	0.00641	4	1	20.66	0
	R15076_4_	K.CWELVE	3597.625	-0.00086	3	1	55.18	0
	R15076_4_	K.CWELVE	3597.625	-0.00086	3	1	105.22	0
	R15076_4_	K.CWELVE	3597.625	-0.00086	3	1	65.57	0
	R15076_4_	K.FHVGPY	2427.129	-0.01688	3	1	65.35	0
	R15076_4_	K.FHVGPY	2427.129	-0.00436	4	1	43.85	0
	R15076_4_	K.FHVGPY	2427.129	-0.00125	3	1	76.81	0
	R15076_4_	K.FHVGPY	2427.129	-0.0041	2	1	154.32	0
	R15076_4_	K.FHVGPY	2427.129	-0.00257	3	1	96.51	0
	R15076_4_	K.FHVGPY	2427.129	-0.0096	4	1	89.06	0
	R15076_4_	K.FHVGPY	2427.129	-0.00434	2	1	154.48	0
	R15076_4_	K.FHVGPY	2427.129	-0.00146	2	1	129.39	0
	R15076_4_	K.FHVGPY	2427.129	-0.00278	3	1	81.91	0
	R15076_4_	K.FHVGPY	2427.129	-0.0055	2	1	140.98	0
	R15076_4_	K.FHVGPY	2427.129	-0.00215	3	1	97.94	0
	R15076_4_	K.FHVGPY	2427.129	-0.0006	2	1	107.77	0
	R15076_4_	K.FHVGPY	2427.129	-0.00154	2	1	160.12	0
	R15076_4_	K.FHVGPY	2427.129	-0.00161	3	1	94.04	0
	R15076_4_	K.FHVGPY	2427.129	-0.00284	3	1	83.19	0
	R15076_4_	K.FHVGPY	2427.129	-0.00116	3	1	92.27	0
	R15076_4_	K.FHVGPY	2427.129	-0.00302	2	1	142.79	0
	R15076_4_	K.FHVGPY	2427.129	0.00166	3	1	50.7	0
	R15076_4_	K.FHVGPY	2427.129	0.00694	3	1	69.74	0
	R15076_4_	K.FHVGPY	2427.129	-0.00416	2	1	95.59	0
	R15076_4_	K.FHVGPY	2427.129	-0.00053	3	1	85.84	0
	R15076_4_	K.FHVGPY	2427.129	-0.00416	2	1	109.16	0
	R15076_4_	K.FHVGPY	2427.129	0.01584	2	1	68.35	0
	R15076_4_	K.FHVGPY	2427.129	0.01312	3	1	68.26	0
	R15076_4_	K.FHVGPY	2427.129	0.01312	3	1	81.1	0
	R15076_4_	K.FHVGPY	2427.129	0.01312	3	1	98.01	0
	R15076_4_	K.FHVGPY	2427.129	0.00082	3	1	81.77	0
	R15076_4_	K.FHVGPY	2427.129	-0.00416	2	1	65.07	0
	R15076_4_	K.FHVGPY	2427.129	0.01312	3	1	81.12	0
	R15076_4_	K.FHVGPY	2427.129	-0.00074	3	1	85.63	0
	R15076_4_	K.FHVGPY	2427.129	-0.0063	2	1	62.54	0
	R15076_4_	K.FHVGPY	2427.129	-0.00119	3	1	91.68	0
	R15076_4_	K.FHVGPY	2427.129	-0.00416	2	1	24.8	0
	R15076_4_	K.FHVGPY	2427.129	0.00118	3	1	94	0
	R15076_4_	K.LCAHPR.	753.3824	0.00018	2	1	33	0
	R15076_4_	K.LIASPR.	656.409	-0.00064	2	1	40.96	0
	R15076_4_	R.AWDDGD	948.3806	-0.01213	2	1	30.38	0
	R15076_4_	R.AWDDGD	948.3806	-0.00131	2	1	41.96	0
	R15076_4_	R.CAVGSII	2643.297	-0.00115	4	1	49.4	0
	R15076_4_	R.CAVGSII	2643.297	0.0043	2	1	24.8	0
	R15076_4_	R.CAVGSII	2643.297	0.00158	3	1	87.97	0
	R15076_4_	R.CAVGSII	2643.297	-0.00058	3	1	20.52	0
	R15076_4_	R.CAVGSII	2643.297	-0.00114	2	1	94.81	0
	R15076_4_	R.CAVGSII	2643.297	-0.00331	4	1	45.69	0
	R15076_4_	R.CAVGSII	2643.297	-0.00138	2	1	111.44	0

R15076_4_R.CAVGSII	2643.297	-0.00307	3	1	74.48	0
R15076_4_R.CAVGSII	2643.297	-0.00203	4	1	70.08	0
R15076_4_R.CAVGSII	2643.297	-0.00356	2	1	115.14	0
R15076_4_R.CAVGSII	2643.297	-0.00307	4	1	48.74	0
R15076_4_R.CAVGSII	2643.297	-0.0025	3	1	22.5	0
R15076_4_R.CAVGSII	2643.297	-0.003	2	1	99.42	0
R15076_4_R.CAVGSII	2643.297	-0.00337	3	1	65.2	0
R15076_4_R.CAVGSII	2643.297	-0.00322	2	1	112.13	0
R15076_4_R.CAVGSII	2643.297	-0.0022	3	1	72.3	0
R15076_4_R.CAVGSII	2643.297	-0.0042	2	1	128.51	0
R15076_4_R.CAVGSII	2643.297	-0.00115	4	1	36.17	0
R15076_4_R.CAVGSII	2643.297	-0.00088	3	1	52.64	0
R15076_4_R.CAVGSII	2643.297	0.0043	2	1	98.36	0
R15076_4_R.CAVGSII	2643.297	-0.00145	3	1	52.02	0
R15076_4_R.CAVGSII	2643.297	0.0043	2	1	60.83	0
R15076_4_R.CAVGSII	2643.297	-0.00019	3	1	51.82	0
R15076_4_R.CAVGSII	2643.297	-0.00115	4	1	29.39	0
R15076_4_R.CAVGSII	2643.297	0.0043	2	1	70.67	0
R15076_4_R.CAVGSII	2643.297	-0.00016	3	1	67.81	0
R15076_4_R.CAVGSII	2643.297	0.0043	2	1	39.13	0
R15076_4_R.CAVGSII	2643.297	0.00158	3	1	65.6	0
R15076_4_R.CAVGSII	2643.297	0.00158	3	1	77.49	0
R15076_4_R.CAVGSII	2643.297	0.00158	3	1	78.86	0
R15076_4_R.CAVGSII	2643.297	-0.00226	3	1	69.11	0
R15076_4_R.CAVGSII	2643.297	-0.00175	3	1	53.54	0
R15076_4_R.ELSTPEI	831.4207	-0.00031	2	1	30.49	0
R15076_4_R.ELSTPEI	1146.527	-0.00163	2	1	21.19	0
R15076_4_R.ETSATPI	1421.666	-0.00158	2	1	50.58	0
R15076_4_R.ETSATPI	1421.666	-0.00718	2	1	52.27	0
R15076_4_R.ETSATPI	1421.666	-0.00312	3	1	40.82	0
R15076_4_R.ETSATPI	1421.666	-0.00188	2	1	51.2	0
R15076_4_R.ETSATPI	1421.666	-0.00718	2	1	51.5	0
R15076_4_R.ETSATPI	1421.666	-0.00718	2	1	36.22	0
R15076_4_R.ETSATPI	1421.666	-0.00718	2	1	30.88	0
R15076_4_R.ETSATPI	1421.666	-0.00718	2	1	43.04	0
R15076_4_R.KLCAHPI	881.4774	0.00001	2	1	36.06	0
R15076_4_R.LEIYQQI	2178.047	-0.00841	3	1	49.96	0
R15076_4_R.LEIYQQI	2178.047	-0.00841	3	1	54.44	0
R15076_4_R.LEIYQQI	2178.047	-0.00568	2	1	48.84	0
R15076_4_R.LEIYQQI	2178.047	-0.00841	3	1	29.31	0
R15076_4_R.LEIYQQI	2178.047	-0.00568	2	1	41.61	0
R15076_4_R.LEIYQQI	2178.047	-0.00841	3	1	53.83	0
R15076_4_R.LEIYQQI	2178.047	-0.00073	3	1	50.27	0
R15076_4_R.LEIYQQI	2178.047	-0.00841	3	1	27.73	0
R15076_4_R.LEIYQQI	2178.047	-0.00841	3	1	25.01	0
R15076_4_R.LEIYQQI	2162.052	-0.00052	2	1	127.54	0
R15076_4_R.LEIYQQI	2162.052	0.00029	3	1	67.86	0
R15076_4_R.LEIYQQI	2162.052	-0.00186	2	1	107.38	0
R15076_4_R.LEIYQQI	2162.052	-0.00042	2	1	141.51	0
R15076_4_R.LEIYQQI	2162.052	-0.00091	3	1	67.8	0
R15076_4_R.LEIYQQI	2162.052	-0.00206	2	1	115.12	0
R15076_4_R.LEIYQQI	2162.052	-0.00148	3	1	71.9	0
R15076_4_R.LEIYQQI	2162.052	-0.00142	2	1	97.42	0
R15076_4_R.LEIYQQI	2162.052	-0.00172	3	1	80.9	0

R15076_4_R.LEIYQQI	2162.052	-0.00172	2	1	101.82	0
R15076_4_R.LEIYQQI	2162.052	-0.00022	3	1	68.52	0
R15076_4_R.LEIYQQI	2162.052	0.00034	2	1	84.9	0
R15076_4_R.LEIYQQI	2162.052	-0.00166	3	1	74.95	0
R15076_4_R.LEIYQQI	2162.052	-0.00194	2	1	82.19	0
R15076_4_R.LEIYQQI	2162.052	-0.00331	3	1	60.13	0
R15076_4_R.LEIYQQI	2162.052	0.00266	3	1	88.16	0
R15076_4_R.LEIYQQI	2162.052	0.00312	2	1	81.06	0
R15076_4_R.LEIYQQI	2162.052	-0.00118	3	1	76.04	0
R15076_4_R.LEIYQQI	2162.052	0.00032	2	1	81.22	0
R15076_4_R.LEIYQQI	2162.052	-0.00106	3	1	75.87	0
R15076_4_R.LEIYQQI	2162.052	0.00218	2	1	93.1	0
R15076_4_R.LEIYQQI	2162.052	-0.0004	3	1	83.33	0
R15076_4_R.LEIYQQI	2162.052	-0.0013	2	1	114.94	0
R15076_4_R.LEIYQQI	2162.052	-0.00277	3	1	71.52	0
R15076_4_R.LEIYQQI	2162.052	-0.0019	2	1	106.86	0
R15076_4_R.LEIYQQI	2162.052	-0.00148	3	1	71.45	0
R15076_4_R.LEIYQQI	2162.052	0.0007	2	1	120.38	0
R15076_4_R.LEIYQQI	2162.052	-0.00163	3	1	81.14	0
R15076_4_R.LEIYQQI	2162.052	-0.00058	3	1	56.55	0
R15076_4_R.LEIYQQI	2162.052	-0.0006	2	1	85.4	0
R15076_4_R.LEIYQQI	2162.052	-0.00118	3	1	62.17	0
R15076_4_R.LEIYQQI	2162.052	-0.00055	3	1	71.41	0
R15076_4_R.LEIYQQI	2162.052	0.00224	2	1	71.18	0
R15076_4_R.LEIYQQI	2162.052	0.00212	2	1	84.79	0
R15076_4_R.LEIYQQI	2162.052	-0.00409	3	1	59.59	0
R15076_4_R.LEIYQQI	2162.052	0.0017	3	1	64.34	0
R15076_4_R.LEIYQQI	2162.052	-0.0006	2	1	102.18	0
R15076_4_R.LEIYQQI	2162.052	-0.0006	2	1	107.5	0
R15076_4_R.NITLAYK	822.472	-0.00041	2	1	38.04	0
R15076_4_R.NITLAYK	822.472	-0.00121	2	1	25.55	0
R15076_4_R.NITLAYK	822.472	-0.00123	2	1	40.16	0
R15076_4_R.NITLAYK	822.472	-0.00171	2	1	30.42	0
R15076_4_R.NITLAYK	822.472	-0.00101	2	1	30.25	0
R15076_4_R.NITLAYK	822.472	-0.00077	2	1	38.17	0
R15076_4_R.NITLAYK	822.472	-0.00077	2	1	38.09	0
R15076_4_R.NITLAYK	822.472	-0.00215	2	1	38.03	0
R15076_4_R.NITLAYK	822.472	-0.00135	2	1	27.71	0
R15076_4_R.NITLAYK	822.472	-0.00015	2	1	38.24	0
R15076_4_R.NITLAYK	822.472	-0.00077	2	1	32.91	0
R15076_4_R.NITLAYK	822.472	-0.00077	2	1	38.08	0
R15076_4_R.NITLAYK	822.472	-0.00077	2	1	28.09	0
R15076_4_R.NITLAYK	822.472	-0.00077	2	1	25.13	0
R15076_4_R.NITLAYK	822.472	-0.00077	2	1	29.14	0
R15076_4_R.NITLAYK	822.472	-0.00077	2	1	42.53	0
R15076_4_R.NITLAYK	822.472	-0.00077	2	1	40.37	0
R15076_4_R.NITLAYK	822.472	-0.00077	2	1	40.34	0
R15076_4_R.NITLAYK	822.472	-0.00077	2	1	21.52	0
R15076_4_R.NITLAYK	822.472	-0.00077	2	1	40.66	0
R15076_4_R.NITLAYK	822.472	-0.00077	2	1	29.33	0
R15076_4_R.NITLAYK	822.472	-0.00077	2	1	23.57	0
R15076_4_R.NITLAYK	822.472	-0.00057	2	1	26.65	0
R15076_4_R.NITLAYK	822.472	-0.00077	2	1	28.64	0
R15076_4_R.NITLAYK	822.472	-0.00077	2	1	30.62	0

R15076_4_	R.QGDFYVF	1181.584	-0.0026	2	1	56.33	0
R15076_4_	R.QGDFYVF	1181.584	0.00827	3	1	27.25	0
R15076_4_	R.QGDFYVF	1181.584	-0.00238	2	1	56.4	0
R15076_4_	R.QGDFYVF	1181.584	-0.00235	3	1	32.15	0
R15076_4_	R.QGDFYVF	1181.584	-0.00242	2	1	66.45	0
R15076_4_	R.QGDFYVF	1181.584	-0.00228	2	1	57.86	0
R15076_4_	R.QGDFYVF	1181.584	-0.0026	2	1	65.58	0
R15076_4_	R.QGDFYVF	1181.584	-0.00024	2	1	64.2	0
R15076_4_	R.QGDFYVF	1181.584	-0.00132	2	1	44.7	0
R15076_4_	R.QGDFYVF	1181.584	-0.009	2	1	24.76	0
R15076_4_	R.QGDFYVF	1181.584	-0.009	2	1	42.47	0
R15076_4_	R.QGDFYVF	1181.584	0.011	2	1	43.77	0
R15076_4_	R.QGDFYVF	1181.584	-0.009	2	1	35.44	0
R15076_4_	R.QGDFYVF	1181.584	-0.009	2	1	44.96	0
R15076_4_	R.QGDFYVF	1181.584	-0.00108	2	1	68.22	0
R15076_4_	R.QGDFYVF	1181.584	-0.009	2	1	53.28	0
R15076_4_	R.QGDFYVF	1181.584	-0.009	2	1	46.56	0
R15076_4_	R.QGDFYVF	1181.584	-0.009	2	1	47.07	0
R15076_4_	R.QGDFYVF	1181.584	-0.009	2	1	28.32	0
R15076_4_	R.QGDFYVF	1181.584	-0.009	2	1	25.97	0
R15076_4_	R.QGDFYVF	1181.584	-0.009	2	1	28.92	0
R15076_4_	R.QGDFYVF	1181.584	-0.009	2	1	43.28	0
R15076_4_	R.QGDFYVF	1181.584	-0.009	2	1	43.76	0
R15076_4_	R.QGDFYVF	1181.584	-0.009	2	1	40.14	0
R15076_4_	R.QGDFYVF	1181.584	-0.009	2	1	45.16	0
R15076_4_	R.QGDFYVF	1696.818	-0.00518	3	1	40.4	0
R15076_4_	R.QGDFYVF	1696.818	0.00496	2	1	57.51	0
R15076_4_	R.RVHPALI	1310.758	-0.00398	3	1	25.1	0
R15076_4_	R.RVHPALI	1310.758	0.00247	3	1	29.66	0
R15076_4_	R.RVHPALI	1310.758	-0.00281	2	1	34.85	0
R15076_4_	R.RVHPALI	1310.758	0.00247	3	1	35.39	0
R15076_4_	R.RVHPALI	1310.758	0.00247	3	1	51.5	0
R15076_4_	R.RVHPALI	1310.758	0.00519	2	1	33.63	0
R15076_4_	R.RVHPALI	1310.758	0.00247	3	1	32.17	0
R15076_4_	R.RVHPALI	1310.758	0.00519	2	1	46.3	0
R15076_4_	R.RVHPALI	1310.758	0.00247	3	1	33.94	0
R15076_4_	R.RVHPALI	1310.758	0.00519	2	1	20.69	0
R15076_4_	R.RVHPALI	1310.758	0.00519	2	1	47.44	0
R15076_4_	R.RVHPALI	1310.758	-0.00206	3	1	36.87	0
R15076_4_	R.RVHPALI	1310.758	-0.00135	2	1	31.56	0
R15076_4_	R.RVHPALI	1310.758	-0.00242	3	1	38.19	0
R15076_4_	R.RVHPALI	1310.758	0.00247	3	1	41.86	0
R15076_4_	R.RVHPALI	1310.758	0.00247	3	1	39.24	0
R15076_4_	R.SEHSYSE	3173.312	-0.00092	3	1	128.49	0
R15076_4_	R.SEHSYSE	3173.312	-0.02309	3	1	26.89	0
R15076_4_	R.SEHSYSE	3173.312	-0.02309	3	1	28.25	0
R15076_4_	R.SEHSYSE	3157.317	0.00472	2	1	61.09	0
R15076_4_	R.SEHSYSE	3157.317	0.00109	3	1	135.16	0
R15076_4_	R.SEHSYSE	3157.317	-0.00134	3	1	113.68	0
R15076_4_	R.SEHSYSE	3157.317	-0.00688	2	1	117.16	0
R15076_4_	R.SEHSYSE	3157.317	-0.00455	3	1	116.37	0
R15076_4_	R.SEHSYSE	3157.317	-0.02073	4	1	83.46	0
R15076_4_	R.SEHSYSE	3157.317	0.00472	2	1	59.54	0
R15076_4_	R.SEHSYSE	3157.317	0.00013	3	1	117.27	0

R15076_4_	R.SEHSYSE	3157.317	0.01927	4	1	57.79	0
R15076_4_	R.SEHSYSE	3157.317	-0.02073	4	1	35.97	0
R15076_4_	R.SEHSYSE	3157.317	-0.00029	3	1	124.28	0
R15076_4_	R.SEHSYSE	3157.317	-0.01528	2	1	89.85	0
R15076_4_	R.SEHSYSE	3157.317	-0.0029	3	1	121.56	0
R15076_4_	R.SEHSYSE	3157.317	-0.01528	2	1	67.26	0
R15076_4_	R.SEHSYSE	3157.317	0.00472	2	1	104.21	0
R15076_4_	R.SEHSYSE	3157.317	0.01927	4	1	51.25	0
R15076_4_	R.SEHSYSE	3157.317	-0.00176	3	1	138.64	0
R15076_4_	R.SEHSYSE	3157.317	0.00472	2	1	107.2	0
R15076_4_	R.SEHSYSE	3157.317	-0.00155	3	1	117.6	0
R15076_4_	R.SEHSYSE	3157.317	0.00337	3	1	119.54	0
R15076_4_	R.SEHSYSE	3157.317	-0.00548	3	1	113.19	0
R15076_4_	R.SIAVYYI	1820.906	-0.00431	2	1	33.46	0
R15076_4_	R.SIAVYYI	1820.906	-0.00076	3	1	43.92	0
R15076_4_	R.SIAVYYI	1820.906	-0.00235	2	1	61.69	0
R15076_4_	R.SIAVYYI	1820.906	0.00104	3	1	78.98	0
R15076_4_	R.SIAVYYI	1820.906	-0.00373	2	1	58.9	0
R15076_4_	R.SIAVYYI	1820.906	-0.00079	3	1	55.46	0
R15076_4_	R.SIAVYYI	1820.906	-0.00379	2	1	53.89	0
R15076_4_	R.SIAVYYI	1820.906	-0.00148	3	1	46.94	0
R15076_4_	R.SIAVYYI	1820.906	-0.00519	2	1	50.98	0
R15076_4_	R.SIAVYYI	1820.906	-0.00274	3	1	58.85	0
R15076_4_	R.SIAVYYI	1820.906	-0.00001	2	1	47.97	0
R15076_4_	R.SIAVYYI	1820.906	0.00104	3	1	49.86	0
R15076_4_	R.SIAVYYI	1820.906	-0.00187	2	1	51.83	0
R15076_4_	R.SIAVYYI	1820.906	-0.00033	2	1	54	0
R15076_4_	R.SIAVYYI	1820.906	0.00339	2	1	50.39	0
R15076_4_	R.SIAVYYI	1820.906	0.00255	2	1	47.99	0
R15076_4_	R.SIAVYYI	1820.906	-0.00623	2	1	58.51	0
R15076_4_	R.SIAVYYI	1820.906	-0.00623	2	1	49.67	0
R15076_4_	R.SIAVYYI	1820.906	0.01377	2	1	38.77	0
R15076_4_	R.SIAVYYI	1820.906	-0.00385	2	1	63.17	0
R15076_4_	R.SIAVYYI	1820.906	-0.00623	2	1	48.72	0
R15076_4_	R.SIAVYYI	1820.906	0.00025	2	1	57.77	0
R15076_4_	R.SIAVYYI	1820.906	-0.00095	2	1	65.67	0
R15076_4_	R.SIAVYYI	1820.906	-0.00145	2	1	32.32	0
R15076_4_	R.SIAVYYI	1820.906	-0.00623	2	1	42.87	0
R15076_4_	R.SIAVYYI	1820.906	-0.00623	2	1	40.14	0
R15076_4_	R.SIAVYYI	1820.906	-0.00623	2	1	43.44	0
R15076_4_	R.VHPALDV	1154.657	0.00409	2	1	31.46	0
R15076_4_	R.VHPALDV	1154.657	-0.00171	3	1	48.38	0
R15076_4_	R.VHPALDV	1154.657	-0.00247	2	1	38.73	0
R15076_4_	R.VHPALDV	1154.657	-0.00084	3	1	47.61	0
R15076_4_	R.VHPALDV	1154.657	-0.00247	2	1	37.06	0
R15076_4_	R.VHPALDV	1154.657	-0.00234	3	1	39.88	0
R15076_4_	R.VHPALDV	1154.657	-0.00253	2	1	34.85	0
R15076_4_	R.VHPALDV	1154.657	-0.00261	3	1	47.8	0
R15076_4_	R.VHPALDV	1154.657	-0.00114	3	1	30.51	0
R15076_4_	R.VHPALDV	1154.657	-0.00169	2	1	33.52	0
R15076_4_	R.VHPALDV	1154.657	-0.00287	2	1	26.4	0
R15076_4_	R.VHPALDV	1154.657	-0.00135	3	1	44.82	0
R15076_4_	R.VHPALDV	1154.657	-0.00269	2	1	34.26	0
R15076_4_	R.VHPALDV	1154.657	-0.00864	3	1	28.11	0

	R15076_4_	R.VHPALDV	1154.657	-0.00864	3	1	42.5	0
	R15076_4_	R.VHPALDV	1154.657	-0.00329	2	1	30.88	0
	R15076_4_	R.VHPALDV	1154.657	0.00015	3	1	44.78	0
	R15076_4_	R.VHPALDV	1154.657	0.00047	2	1	37.33	0
	R15076_4_	R.VHPALDV	1154.657	-0.00864	3	1	47.89	0
	R15076_4_	R.VHPALDV	1154.657	0.00097	2	1	27.24	0
	R15076_4_	R.VHPALDV	1154.657	-0.00864	3	1	39.97	0
	R15076_4_	R.VHPALDV	1154.657	-0.00864	3	1	41.16	0
	R15076_4_	R.VHPALDV	1154.657	0.00409	2	1	30.13	0
	R15076_4_	R.VHPALDV	1154.657	-0.00864	3	1	44.79	0
	R15076_4_	R.VHPALDV	1154.657	0.00009	2	1	44.54	0
	R15076_4_	R.VHPALDV	1154.657	-0.00864	3	1	45.48	0
	R15076_4_	R.VHPALDV	1154.657	-0.00864	3	1	38.68	0
	R15076_4_	R.VHPALDV	1154.657	-0.00117	2	1	21.22	0
	R15076_4_	R.VHPALDV	1154.657	-0.00042	3	1	47.8	0
	R15076_4_	R.VHPALDV	1154.657	-0.00864	3	1	42.23	0
	R15076_4_	R.VHPALDV	1154.657	-0.00864	3	1	42.31	0
	R15076_4_	R.VHPALDV	1154.657	-0.00864	3	1	38.74	0
	R15076_4_	R.VHPALDV	1154.657	-0.00864	3	1	43.98	0
$2-1	sp|P69897	18	13	40.09%	49670.28	4.78		
	R15076_4_	K.EVDEQM*	1462.684	-0.00851	2	1	34.71	0
	R15076_4_	K.EVDEQMI	1446.689	-0.00343	2	1	46.08	0
	R15076_4_	K.IREEYPI	1077.532	-0.00313	3	1	33.59	0
	R15076_4_	K.LAVNM*V	1159.629	-0.0028	2	1	27.72	0
	R15076_4_	K.LAVNMVF	1143.634	-0.00016	2	1	56.59	0
	R15076_4_	K.LTTPTYC	2708.338	0.00279	3	1	28.22	0
	R15076_4_	K.NMM*AAC	1081.422	-0.00217	2	1	37.23	0
	R15076_4_	K.NMMAACI	1065.427	-0.00201	2	1	40.18	0
	R15076_4_	K.NSSYFVE	1696.833	0.00021	2	1	50.35	0
	R15076_4_	R.AILVDLE	1631.831	-0.00182	2	1	36.34	0
	R15076_4_	R.ALTVPEI	1659.895	-0.00209	2	1	47.05	0
	R15076_4_	R.FPGQLNA	1130.595	0.00255	2	1	52.18	0
	R15076_4_	R.FPGQLNA	1130.595	0.00119	2	1	51.96	0
	R15076_4_	R.FPGQLNA	1130.595	0.00255	2	1	40.76	0
	R15076_4_	R.IMNTFSV	1319.703	-0.00994	2	1	77.19	0
	R15076_4_	R.ISEQFTA	1245.593	-0.00279	2	1	34.95	0
	R15076_4_	R.ISVYYNE	1301.637	0.00446	2	1	70.26	0
	R15076_4_	R.SGPFGQI	2798.343	0.00795	3	1	21.04	0
$3-1	sp|Q3KRE8	18	13	39.78%	49952.54	4.78		
	R15076_4_	K.EVDEQM*	1462.684	-0.00851	2	1	34.71	0
	R15076_4_	K.EVDEQMI	1446.689	-0.00343	2	1	46.08	0
	R15076_4_	K.IREEYPI	1077.532	-0.00313	3	1	33.59	0
	R15076_4_	K.LAVNM*V	1159.629	-0.0028	2	1	27.72	0
	R15076_4_	K.LAVNMVF	1143.634	-0.00016	2	1	56.59	0
	R15076_4_	K.LTTPTYC	2708.338	0.00279	3	1	28.22	0
	R15076_4_	K.MSATFIC	1857.942	-0.00109	2	1	61.18	0
	R15076_4_	K.NMM*AAC	1081.422	-0.00217	2	1	37.23	0
	R15076_4_	K.NMMAACI	1065.427	-0.00201	2	1	40.18	0
	R15076_4_	K.NSSYFVE	1696.833	0.00021	2	1	50.35	0
	R15076_4_	R.AILVDLE	1631.831	-0.00182	2	1	36.34	0
	R15076_4_	R.FPGQLNA	1130.595	0.00255	2	1	52.18	0
	R15076_4_	R.FPGQLNA	1130.595	0.00119	2	1	51.96	0
	R15076_4_	R.FPGQLNA	1130.595	0.00255	2	1	40.76	0
	R15076_4_	R.INVYYNE	1385.67	-0.00319	2	1	56.44	0

	R15076_4_R.ISEQFTA	1245.593	-0.00279		2	1	34.95	0
	R15076_4_R.SGPFGQI	2798.343	0.00795		3	1	21.04	0
	R15076_4_R.YLTVAAI	1053.609	-0.0036		2	1	45.97	0
$4-1	tr|G3V7C6	16	11	34.38%	49830.48	4.79		
	R15076_4_K.EVDEQM*	1462.684	-0.00851		2	1	34.71	0
	R15076_4_K.EVDEQMI	1446.689	-0.00343		2	1	46.08	0
	R15076_4_K.IREEYPI	1077.532	-0.00313		3	1	33.59	0
	R15076_4_K.LAVNM*V	1159.629	-0.0028		2	1	27.72	0
	R15076_4_K.LAVNMVI	1143.634	-0.00016		2	1	56.59	0
	R15076_4_K.LTTPTYC	2708.338	0.00279		3	1	28.22	0
	R15076_4_K.MSATFIC	1857.942	-0.00109		2	1	61.18	0
	R15076_4_K.NMM*AAC	1081.422	-0.00217		2	1	37.23	0
	R15076_4_K.NMMAACI	1065.427	-0.00201		2	1	40.18	0
	R15076_4_K.NSSYFVI	1696.833	0.00021		2	1	50.35	0
	R15076_4_R.FPGQLNA	1130.595	0.00255		2	1	52.18	0
	R15076_4_R.FPGQLNA	1130.595	0.00119		2	1	51.96	0
	R15076_4_R.FPGQLNA	1130.595	0.00255		2	1	40.76	0
	R15076_4_R.IMNTFSV	1319.703	-0.00994		2	1	77.19	0
	R15076_4_R.ISEQFTA	1245.593	-0.00279		2	1	34.95	0
	R15076_4_R.SGPFGQI	2798.343	0.00795		3	1	21.04	0
$5-1	sp|P68370	13	10	29.49%	50135.07	4.94		
$5-2	sp|Q6P9V9	13	10	29.49%	50151.07	4.94		
	R15076_4_K.DVNAAIA	1015.578	-0.00145		2	1	52.62	0
	R15076_4_K.EIIDLVI	1085.62	-0.00186		2	1	57.16	0
	R15076_4_K.EIIDLVI	1085.62	-0.0014		2	1	49.24	0
	R15076_4_K.FDLMYAK	887.4331	0.00041		2	1	33.18	0
	R15076_4_K.TIGGGDI	2007.893	-0.00237		3	1	45.4	0
	R15076_4_K.VGINYQF	1824.985	-0.00202		2	1	34.57	0
	R15076_4_R.AVFVDLI	1701.906	-0.00035		2	1	89.94	0
	R15076_4_R.FDGALNV	2409.209	0.01578		2	1	34.11	0
	R15076_4_R.LSVDYGK	781.409	-0.00094		2	1	46.26	0
	R15076_4_R.NLDIERI	1718.882	-0.00402		3	1	33.79	0
	R15076_4_R.NLDIERI	1718.882	-0.01074		2	1	62.82	0
	R15076_4_R.QLFHPEC	1410.774	-0.00002		2	1	69.92	0
	R15076_4_R.QLFHPEC	1410.774	-0.00112		3	1	39.82	0
$6-1	sp|Q4QRB4	13	8	20.44%	50418.13	4.82		
	R15076_4_K.LAVNM*V	1159.629	-0.0028		2	1	27.72	0
	R15076_4_K.LAVNMVI	1143.634	-0.00016		2	1	56.59	0
	R15076_4_K.NMM*AAC	1081.422	-0.00217		2	1	37.23	0
	R15076_4_K.NMMAACI	1065.427	-0.00201		2	1	40.18	0
	R15076_4_K.NSSYFVI	1696.833	0.00021		2	1	50.35	0
	R15076_4_R.AILVDLI	1631.831	-0.00182		2	1	36.34	0
	R15076_4_R.FPGQLNA	1130.595	0.00255		2	1	52.18	0
	R15076_4_R.FPGQLNA	1130.595	0.00119		2	1	51.96	0
	R15076_4_R.FPGQLNA	1130.595	0.00255		2	1	40.76	0
	R15076_4_R.IMNTFSV	1319.703	-0.00994		2	1	77.19	0
	R15076_4_R.ISEQFTA	1245.593	-0.00279		2	1	34.95	0
	R15076_4_R.ISVYYNI	1397.67	-0.00319		2	1	64.33	0
	R15076_4_R.ISVYYNI	1397.67	0.00409		3	1	52.12	0
$7-1	tr|Q4KM71	9	7	10.01%	75485.88	9.45		
$7-2	tr|F1LQW3	9	7	11.55%	65695.05	8.87		
	R15076_4_K.AELDDTI	1047.478	0.0048		2	1	35.84	0
	R15076_4_K.ANLSLLI	786.4832	-0.00954		2	1	49.77	0
	R15076_4_K.ISDSEGI	882.4203	-0.01239		2	1	27.96	0

	R15076_4_R.AVVIVDI	886.4993	-0.01346		2	1	60.36	0
	R15076_4_R.AVVIVDI	886.4993	0.00654		2	1	42.16	0
	R15076_4_R.FATHAAA	1143.627	-0.00582		2	1	47.9	0
	R15076_4_R.FATHAAA	1143.627	0.00146		3	1	51.47	0
	R15076_4_R.LFVGNLI	1807.911	-0.00147		2	1	70.93	0
	R15076_4_R.MEELHSG	1389.614	-0.01177		3	1	20.25	0
$8-1	sp|Q6AY56	8	6	19.60%	50036.93	4.98		
	R15076_4_K.FDLMYAK	887.4331	0.00041		2	1	33.18	0
	R15076_4_K.VGINYQF	1824.985	-0.00202		2	1	34.57	0
	R15076_4_R.FDGALNV	2409.209	0.01578		2	1	34.11	0
	R15076_4_R.GHYTVGK	1801.944	-0.00116		3	1	23.04	0
	R15076_4_R.NLDIERI	1718.882	-0.00402		3	1	33.79	0
	R15076_4_R.NLDIERI	1718.882	-0.01074		2	1	62.82	0
	R15076_4_R.QLFHPEG	1410.774	-0.00002		2	1	69.92	0
	R15076_4_R.QLFHPEG	1410.774	-0.00112		3	1	39.82	0
$9-1	tr|M0RBS9	6	6	12.24%	60984.15	5.95		
$9-2	sp|Q8K4V4	6	6	12.24%	61014.18	5.95		
	R15076_4_K.NSTTDQV	1509.754	0.00162		2	1	50.77	0
	R15076_4_K.SESGYGI	1115.512	0.00507		2	1	22.18	0
	R15076_4_R.EFANFTI	1128.547	-0.00545		2	1	28.68	0
	R15076_4_R.GLEEYLI	980.4935	0.00074		2	1	28.85	0
	R15076_4_R.GQVSEGG	1030.528	-0.00512		2	1	45.83	0
	R15076_4_R.ILEVNGV	1579.844	-0.00888		2	1	83.84	0
$10-1	sp|Q5FVM4	7	5	11.13%	54924.67	9.01		
	R15076_4_K.VELDNMI	1086.561	-0.0018		2	1	35.57	0
	R15076_4_R.AVVIVDI	886.4993	-0.01346		2	1	60.36	0
	R15076_4_R.AVVIVDI	886.4993	0.00654		2	1	42.16	0
	R15076_4_R.FACHSAS	1248.615	0.00265		2	1	61.83	0
	R15076_4_R.FACHSAS	1248.615	-0.0024		3	1	47.07	0
	R15076_4_R.FAQPGSI	1695.747	-0.00558		2	1	45.7	0
	R15076_4_R.MEELHNG	1384.653	-0.00022		2	1	72.8	0
$11-1	sp|P62630	6	5	14.50%	50113.26	9.1		
$11-2	tr|M0R757	6	5	14.50%	50107.29	9.1		
	R15076_4_K.DGSASGT	2994.556	-0.00899		3	1	58.13	0
	R15076_4_K.QLIVGVN	870.5407	-0.00176		2	1	23.05	0
	R15076_4_K.STTTGHI	1120.6	-0.00139		2	1	47.36	0
	R15076_4_K.YYVTIII	1404.727	0.01157		3	1	37.9	0
	R15076_4_K.YYVTIII	1404.727	-0.00571		2	1	55.64	0
	R15076_4_R.QTVAVGV	914.567	-0.00577		2	1	55.82	0
$12-1	tr|G3V908	5	5	9.40%	57608.89	8.04		
	R15076_4_K.FASFIDK	827.4298	-0.00295		2	1	35.7	0
	R15076_4_K.LALDIEI	1277.71	-0.00278		2	1	61.28	0
	R15076_4_K.LLEGEEG	1005.467	-0.00226		2	1	47.66	0
	R15076_4_R.FLEQQNK	1476.806	0.01292		2	1	36.02	0
	R15076_4_R.SLDLDSI	1302.715	-0.00026		2	1	66.76	0
$13-1	sp|P48721	5	5	9.87%	73856.85	5.97		
$13-2	tr|F1M953	5	5	9.87%	73743.69	5.87		
	R15076_4_K.STNGDTI	2055.962	-0.01092		2	1	53.37	0
	R15076_4_K.VLENAEG	958.4952	0.00216		2	1	53.5	0
	R15076_4_K.VQQTVQI	1290.68	0.00737		2	1	66.55	0
	R15076_4_R.AQFEGIV	1333.736	-0.0032		2	1	43.44	0
	R15076_4_R.VINEPTA	1645.88	0.0068		2	1	42.01	0
$14-1	sp|P63018	5	5	9.60%	70870.22	5.37		
$14-2	tr|M0RCB1	5	5	9.67%	70428.7	5.43		

	R15076_4_K.DAGTIAC		1199.674	0.00153	2	1	33.03	0
	R15076_4_K.LLQDFFN		1081.568	-0.00505	2	1	35.99	0
	R15076_4_K.NQVAMNF		1649.795	-0.00289	2	1	36.48	0
	R15076_4_R.FEELNAI		1253.616	-0.00233	2	1	51.37	0
	R15076_4_R.IINEPTA		1659.895	0.00027	2	1	89.88	0
$15-1	tr|F1M1DC	5	4	7.77%	57308.33	7.6		
	R15076_4_K.FASFIDK		827.4298	-0.00295	2	1	35.7	0
	R15076_4_K.NKYEDEI		1308.654	-0.00124	3	1	45.87	0
	R15076_4_K.NKYEDEI		1308.654	0.00148	2	1	51.42	0
	R15076_4_R.FLEQQNK		1476.806	0.01292	2	1	36.02	0
	R15076_4_R.SLDLDSI		1302.715	-0.00026	2	1	66.76	0
$16-1	tr|M0R3X6	5	4	18.05%	30610.76	6.97		
$16-2	sp|P47727	5	4	18.05%	30577.76	8.21		
	R15076_4_K.ILLNACC		1458.734	0.00171	2	1	63.26	0
	R15076_4_K.QLQTEGI		1128.601	-0.00251	2	1	53.23	0
	R15076_4_R.FHQLDII		1582.797	0.00178	3	1	36.49	0
	R15076_4_R.FHQLDII		1582.797	0.0045	2	1	27.51	0
	R15076_4_R.SETITEI		1692.836	-0.00173	2	1	80.72	0
$17-1	sp|P15205	4	4	2.68%	269496.5	4.74		
$17-2	tr|F1LRL9	4	4	2.68%	269639.6	4.74		
	R15076_4_K.NLISPDI		2081.164	-0.00862	2	1	66.38	0
	R15076_4_K.TTEAAAT		2256.183	0.00755	3	1	24.96	0
	R15076_4_R.AVLDALI		1028.599	0.00589	2	1	38.75	0
	R15076_4_R.HNLQDFI		1241.664	0.01095	2	1	38.44	0
$18-1	sp|P97576	4	3	17.05%	24296.74	8.57		
	R15076_4_K.FDPYEHE		2015.95	-0.00779	3	1	23.62	0
	R15076_4_K.FDPYEHE		2015.95	0.01159	4	1	36.59	0
	R15076_4_R.ALADTEN		1002.521	-0.0113	2	1	63.69	0
	R15076_4_R.TLRPALV		1152.746	-0.00642	2	1	61.41	0
$19-1	sp|P01946	3	3	32.39%	15328.35	7.81		
$19-2	tr|B1H216	3	3	32.39%	15328.35	7.81		
	R15076_4_K.AADHVEI		2297.152	-0.00341	3	1	46.84	0
	R15076_4_K.IGGHGGE		1572.74	0.00737	2	1	48.75	0
	R15076_4_R.MFAAFPT		1013.512	-0.00026	2	1	46.76	0
$20-1	sp|P11517	3	3	21.77%	15982.24	8.91		
	R15076_4_K.LHVDPEN		1126.564	-0.00875	2	1	35.99	0
	R15076_4_K.VINAFNI		1090.589	-0.0036	2	1	56.01	0
	R15076_4_K.VNADNVC		1285.649	-0.00324	2	1	89.58	0
$21-1	sp|Q63716	3	3	12.06%	22109.14	8.27		
	R15076_4_K.DISLSDY		940.4622	-0.00122	2	1	37.68	0
	R15076_4_K.IGHPAPS		953.5203	0.00487	3	1	20.71	0
	R15076_4_R.SVDEILF		831.457	-0.0013	2	1	29.12	0
$22-1	sp|Q9JJ54	3	3	8.22%	38217.52	7.61		
	R15076_4_K.IFVGGLS		1488.758	-0.00469	2	1	46.75	0
	R15076_4_K.LDPITGF		771.4359	0.0032	2	1	29.41	0
	R15076_4_R.GFGFVLI		914.5135	0.00075	2	1	28.66	0
$23-1	tr|V9GZ85	3	3	8.82%	41619.23	5.46		
$23-2	tr|D3ZRN3	3	3	8.78%	41962.62	5.3		
$23-3	sp|P60711	3	3	8.80%	41736.29	5.29		
$23-4	sp|P63259	3	3	8.80%	41792.4	5.31		
	R15076_4_K.CDVDIR.		777.356	0.00325	2	1	30.17	0
	R15076_4_K.SYELPDC		1790.892	-0.00082	2	1	68.83	0
	R15076_4_R.HQGVMVC		1171.571	-0.00207	2	1	51.58	0
$24-1	tr|D3ZH98	3	2	6.10%	68856.3	5.54		

	R15076_4_K.NINADEA	2261.141	-0.01195		2	1	23.47	0
	R15076_4_K.NINADEA	2261.141	-0.01467		3	1	30.72	0
	R15076_4_K.NQVAMNI	1649.795	-0.00289		2	1	36.48	0
$25-1	tr|D3ZN95	2	2	1.03%	209150.3	6.57		
	R15076_4_R.ESHTAVV	1263.622	-0.01118		2	1	46.39	0
	R15076_4_R.GPFSEIS	1082.552	0.00036		2	1	51.09	0
$26-1	tr|D3ZPW4	2	2	1.80%	125018.8	8.98		
	R15076_4_K.LGGIGVE	1654.916	-0.01644		2	1	22.4	0
	R15076_4_R.VIDLR.A	615.3824	-0.00038		2	1	28.55	0
$27-1	tr|M0R584	2	2	10.41%	29118.74	6.6		
$27-2	tr|F7FEZ6	2	2	8.75%	34222.82	9.27		
$27-3	tr|Q6P6G9	2	2	8.95%	33619.24	9.27		
$27-4	tr|Q5I0M7	2	2	8.75%	34195.8	9.27		
$27-5	sp|P04256	2	2	8.75%	34211.86	9.2		
	R15076_4_R.EDSQRPC	1437.744	-0.00826		2	1	41.49	0
	R15076_4_R.GFAFVTF	1699.76	0.00716		2	1	38.96	0
$28-1	sp|P00762	2	2	10.57%	25959.22	4.71		
	R15076_4_K.SRIQVRI	2950.549	0.05113		4	1	36.7	0
	R15076_4_R.LGEHNIN	2211.104	-0.00424		2	1	88.19	0
$29-1	sp|P04905	2	2	11.47%	25913.66	8.27		
$29-2	tr|B6DYQ1	2	2	11.47%	25968.78	8.78		
$29-3	tr|Q6LDP3	2	2	11.57%	25685.34	8.27		
$29-4	tr|G3V983	2	2	11.47%	25894.61	7.67		
	R15076_4_K.CLDAFPN	1077.54	0.00059		2	1	28.08	0
	R15076_4_K.LGLDFPN	1789.948	0.00066		2	1	56.62	0
$30-1	sp|P06761	2	2	2.60%	72346.1	5.07		
	R15076_4_R.IINEPTA	1659.895	0.00027		2	1	89.88	0
	R15076_4_R.IINEPTA	1815.996	0.00355		2	1	52.74	0
$31-1	sp|P35704	2	2	9.09%	21783.41	5.34		
	R15076_4_K.SLSQNYC	1108.6	-0.00228		2	1	48.53	0
	R15076_4_R.GLFIIDA	876.5189	0.00619		2	1	40.59	0
$32-1	tr|Q0QEU1	2	2	15.13%	25535.14	8.84		
$32-2	sp|P04797	2	2	10.81%	35827.59	8.14		
$32-3	tr|D3ZGY4	2	2	10.81%	35822.57	7.63		
$32-4	tr|M0R590	2	2	10.81%	35813.56	8.14		
	R15076_4_K.WGDAGAE	2277.038	-0.01472		2	1	68.15	0
	R15076_4_R.GAAQNII	1369.743	-0.00407		2	1	45.3	0
$33-1	tr|Q64715	2	2	1.21%	198564.1	4.76		
$33-2	tr|F1MAQ5	2	2	1.20%	198902.4	4.75		
$33-3	tr|F1LNK0	2	2	1.18%	202242.2	4.79		
$33-4	sp|P15146	2	2	1.18%	202408.3	4.77		
	R15076_4_R.LINQPLF	1150.683	-0.00075		2	1	44.25	0
	R15076_4_R.SILTEQI	1371.773	0.00022		2	1	32.3	0
$34-1	tr|Q6AYI1	2	2	3.74%	69238.36	9.06		
$34-2	tr|B6DTP5	2	2	3.75%	69111.22	9.01		
	R15076_4_R.ELAQQVG	1792.865	0.01191		2	1	48.03	0
	R15076_4_R.QLAEDFI	963.5146	0.00183		2	1	50.84	0
$35-1	sp|Q9JLT0	2	2	1.11%	228962.5	5.49		
$35-2	tr|G3V9Y1	2	2	1.11%	228995.5	5.44		
	R15076_4_K.KQELEEI	1738.897	0.00431		3	1	30.75	0
	R15076_4_R.DRAEAEA	917.4435	0.00055		2	1	21.32	0
$36-1	tr|D4A8X8	10	1	0.94%	70075.66	7.19		
	R15076_4_R.DFETLK.	752.3825	-0.00111		2	1	30.66	0
	R15076_4_R.DFETLK.	752.3825	0.00049		2	1	35.17	0

	R15076_4_	R.DFETLK.	752.3825	-0.00123	2	1	32.69	0
	R15076_4_	R.DFETLK.	752.3825	-0.01023	2	1	34.84	0
	R15076_4_	R.DFETLK.	752.3825	-0.00103	2	1	37.27	0
	R15076_4_	R.DFETLK.	752.3825	-0.01023	2	1	38.1	0
	R15076_4_	R.DFETLK.	752.3825	-0.01023	2	1	37.21	0
	R15076_4_	R.DFETLK.	752.3825	-0.01023	2	1	32.85	0
	R15076_4_	R.DFETLK.	752.3825	0.00977	2	1	37.28	0
	R15076_4_	R.DFETLK.	752.3825	0.00977	2	1	24.72	0
$37-1	tr|D4A4U8	3	1	2.14%	47465.31	5.91		
	R15076_4_	K.ELALKNF	1026.594	0.00575	2	1	22.9	0
	R15076_4_	K.ELALKNF	1026.594	0.00145	2	1	20.54	0
	R15076_4_	K.ELALKNF	1026.594	0.00145	2	1	23.61	0
$38-1	tr|M0RA91	3	1	10.67%	8182.27	8		
	R15076_4_	K.LVKENNI	985.5789	0.00615	2	1	22.33	0
	R15076_4_	K.LVKENNI	985.5789	0.00615	2	1	20.16	0
	R15076_4_	K.LVKENNI	985.5789	0.00615	2	1	22.11	0
$39-1	tr|Q4KLM0	3	1	1.55%	43348.13	9.7		
	R15076_4_	K.IPPFEK.	730.4134	-0.00468	2	1	23.26	0
	R15076_4_	K.IPPFEK.	730.4134	-0.00414	2	1	20.01	0
	R15076_4_	K.IPPFEK.	730.4134	-0.00428	2	1	20.11	0
$40-1	tr|D3ZB99	2	1	0.50%	136011.2	5.48		
	R15076_4_	R.LNENLK.	730.4094	-0.00886	2	1	22.11	0
	R15076_4_	R.LNENLK.	730.4094	-0.00834	2	1	20.97	0
$41-1	tr|D4A7T9	2	1	1.60%	92801.57	5.31		
	R15076_4_	K.SNSDLQV	1532.766	-0.01915	3	1	27	0
	R15076_4_	K.SNSDLQV	1532.766	-0.01915	3	1	24.62	0
$42-1	sp|Q8VIP2	2	1	1.50%	94920.14	8.35		
$42-2	tr|C7C5T1	2	1	1.50%	95064.31	8.35		
	R15076_4_	R.ALADLSV	1395.795	0.01725	4	1	21.85	0
	R15076_4_	R.ALADLSV	1395.795	0.01725	4	1	22.58	0
$43-1	tr|A0JN13	1	1	8.09%	19791.9	5.78		
	R15076_4_	R.TVLDSGI	1533.787	-0.01872	3	1	22.12	0
$44-1	tr|A1A5R0	1	1	1.60%	58217.5	8.89		
$44-2	tr|F1LPJ8	1	1	1.60%	58086.31	8.89		
	R15076_4_	R.NLTAIGY	879.4934	0.0007	2	1	23.56	0
$45-1	tr|A1L1J7	1	1	2.08%	37569.31	4.94		
	R15076_4_	K.GDFETIK	809.404	-0.00876	2	1	24.49	0
$46-1	tr|A5I9F0	1	1	0.49%	162181.4	5.69		
$46-2	tr|Q9WUL3	1	1	1.39%	56157.97	5.69		
$46-3	tr|F1LPL4	1	1	0.66%	119467.7	5.84		
	R15076_4_	K.IAEIQAF	800.4625	-0.01028	2	1	22.42	0
$47-1	tr|B0BN75	1	1	1.89%	46939.17	8.85		
	R15076_4_	K.LLKEQEE	1044.568	-0.01887	2	1	24.2	0
$48-1	sp|B0BNK9	1	1	2.04%	44552.44	5.21		
	R15076_4_	K.LLGSSHF	897.5265	0.01373	2	1	28.38	0
$49-1	tr|B0BNM8	1	1	3.30%	24169.27	5.65		
	R15076_4_	K.IQM*KME	923.4689	-0.00387	2	1	27.48	0
$50-1	sp|B1WBT0	1	1	0.97%	71913.06	6.35		
$50-2	sp|Q5U2W6	1	1	1.44%	46848.01	10.8		
	R15076_4_	K.DM*ETLK	752.3495	-0.00325	2	1	30.49	0
$51-1	tr|B1WBX7	1	1	5.42%	18663.96	4.88		
$51-2	tr|G3V8Q1	1	1	5.45%	18507.77	4.78		
	R15076_4_	R.DSIVLEI	1059.568	0.01533	2	1	20.15	0
$52-1	tr|B2GUW2	1	1	1.27%	79868.7	6.86		

	R15076_4_K.SLPLISA	943.5571	0.00436	2	1	31.7	0	
$53-1	tr|B6DYQ7	1	1	5.24%	23438.61	6.89		
$53-2	tr|D4A8S2	1	1	6.32%	20065.23	7		
$53-3	sp|P04906	1	1	5.24%	23438.61	6.89		
	R15076_4_R.MLLADQC	1276.635	-0.00153	2	1	26.13	0	
$54-1	tr|C0LP03	1	1	0.70%	98052.06	6.79		
$54-2	tr|G3V6F8	1	1	0.69%	99623.93	6.99		
	R15076_4_R.LFETLK.	750.4396	-0.00064	2	1	31.13	0	
$55-1	tr|C9WPN6	1	1	11.29%	13636.84	9.92		
$55-2	tr|W8CEN7	1	1	2.97%	51153.05	8.76		
$55-3	sp|P81795	1	1	2.97%	51078.88	8.66		
	R15076_4_K.IVLTNPV	1558.815	0.0018	2	1	29.31	0	
$56-1	tr|D3Z8BC	1	1	6.60%	12252.19	9.82		
	R15076_4_M.VAPAVLF	725.4668	0.00014	2	1	22.57	0	
$57-1	tr|D3Z954	1	1	1.34%	59219.92	6.81		
	R15076_4_K.RM*FLLA	922.5291	-0.00363	2	1	21.97	0	
$58-1	tr|D3ZE49	1	1	0.75%	87545.83	4.66		
$58-2	tr|M0R78C	1	1	1.03%	63199.99	4.49		
	R15076_4_K.DSLVPR.	686.3832	-0.0002	2	1	31.82	0	
$59-1	tr|D3ZFN9	1	1	0.53%	150044	6.08		
	R15076_4_K.LQLEKRF	942.5843	0.01156	2	1	22.98	0	
$60-1	tr|D3ZFQ7	1	1	3.97%	28377.65	9.68		
	R15076_4_R.QPLNSIC	1182.695	0.00925	2	1	30.88	0	
$61-1	tr|D3ZG47	1	1	0.91%	87759.15	8.69		
$61-2	tr|D4A2P1	1	1	0.61%	132151.8	5.74		
	R15076_4_K.LQGERKK	858.5156	0.00282	2	1	20.39	0	
$62-1	tr|D3ZH11	1	1	0.39%	145991.7	6.58		
$62-2	tr|D3ZH16	1	1	0.39%	145260.3	8.8		
$62-3	tr|F1M841	1	1	0.75%	75456.28	8.92		
$62-4	tr|Q4V8M7	1	1	0.64%	88403.05	5.74		
$62-5	tr|B2GV26	1	1	0.42%	135814.5	6.22		
$62-6	tr|D3ZFT5	1	1	0.63%	90730.64	8.49		
$62-7	tr|Q5Y9B8	1	1	0.39%	146103	6.2		
$62-8	tr|F1M6I1	1	1	0.70%	79322.34	7.53		
$62-9	tr|A0A096	1	1	0.70%	81716.7	5.72		
$62-10	tr|D3ZRJ5	1	1	0.40%	142783.6	8.84		
$62-11	tr|E9PTA9	1	1	0.62%	91364.7	5.87		
$62-12	tr|F1LR97	1	1	0.64%	88299.94	5.82		
$62-13	tr|Q5Y9B7	1	1	0.62%	91377.74	5.92		
$62-14	tr|G3V905	1	1	0.39%	146090	6.16		
$62-15	tr|D3Z8H5	1	1	0.39%	146956.3	6.12		
$62-16	tr|A0A0AC	1	1	0.97%	57687.6	5.32		
$62-17	sp|Q6MG97	1	1	1.10%	50766.7	4.97		
	R15076_4_R.CLFGK.I	624.3174	-0.00137	2	1	22.3	0	
$63-1	tr|D3ZH42	1	1	0.48%	138350.5	6.34		
	R15076_4_K.VSNGLK.	617.3617	-0.01103	2	1	24.28	0	
$64-1	tr|D3ZHF9	1	1	0.42%	189941.8	6.55		
$64-2	tr|A0A096	1	1	0.53%	150928.7	7.3		
	R15076_4_K.LSPQGKF	785.4628	0.01007	2	1	33.09	0	
$65-1	tr|D3ZI44	1	1	3.70%	26668.24	10.9		
	R15076_4_R.EASTLRA	1004.537	-0.01566	2	1	24.53	0	
$66-1	tr|D3ZNQ2	1	1	2.63%	37094.97	4.92		
	R15076_4_K.GLPELM*	1090.575	-0.01073	3	1	20.63	0	
$67-1	tr|D3ZPP5	1	1	1.81%	59606.57	7.45		

	R15076_4_R.VVVGEPI	1024.567	0.01189	3	1	21.8	0		
$68-1	tr|D3ZRE3	1	1	5.75%	41932.8	9.62			
$68-2	tr|A0JPL2	1	1	6.23%	38914.42	9.59			
	R15076_4_K.AAQQAAS	2001.958	0.00298	3	1	21.33	0		
$69-1	tr|D3ZXS4	1	1	2.10%	38715.16	5.63			
	R15076_4_K.HHEDVKI	920.4697	-0.01445	2	1	20.31	0		
$70-1	tr|D3ZYM5	1	1	0.89%	85767.15	7.24			
	R15076_4_K.VANQLDK	787.4308	-0.00189	2	1	22.31	0		
$71-1	tr|D4A0G9	1	1	0.57%	138172.6	5			
	R15076_4_K.ENVVVYI	878.473	-0.00155	2	1	24.23	0		
$72-1	tr|D4A1X2	1	1	0.68%	100717.4	7.83			
$72-2	tr|D4A2C3	1	1	0.70%	97914.28	7.84			
$72-3	tr|M0R8N6	1	1	0.85%	80720.22	5.86			
	R15076_4_R.EMPLLK.	730.4168	-0.00026	2	1	20.11	0		
$73-1	tr|D4A3X1	1	1	1.25%	42680.93	4.86			
	R15076_4_R.CAHPR.(	640.2984	0.00566	2	1	32.21	0		
$74-1	tr|D4A5W5	1	1	0.58%	134674	8.86			
	R15076_4_R.LATVRAI	786.4944	0.0017	2	1	21.05	0		
$75-1	tr|D4A6C6	1	1	1.59%	54600.42	5.31			
	R15076_4_R.VTNFVAC	934.5105	-0.00151	2	1	21.25	0		
$76-1	tr|D4A7X9	1	1	1.00%	125317.3	9.25			
	R15076_4_K.CVYVIGI	1350.756	0.00348	2	1	22.22	0		
$77-1	tr|D4A961	1	1	2.61%	33548.44	9.12			
	R15076_4_R.GASAMLI	818.4553	0.00253	2	1	33.39	0		
$78-1	tr|D4A9Q5	1	1	2.26%	50319.91	8.05			
	R15076_4_K.EHRVGII	1211.653	-0.01233	3	1	25.46	0		
$79-1	tr|D4AA10	1	1	0.92%	73235.73	9.15			
$79-2	sp|A2VD13	1	1	0.87%	77120.26	9.08			
	R15076_4_R.LQPDIK.	713.4192	-0.00046	2	1	23.26	0		
$80-1	tr|D4AAW3	1	1	1.04%	78321.42	8.05			
$80-2	tr|F1LZH7	1	1	0.49%	166665.5	8.71			
	R15076_4_K.YQDFLSI	900.4462	-0.01118	2	1	30.14	0		
$81-1	tr|D4ADE4	1	1	0.52%	108886.9	4.92			
$81-2	sp|Q9JKB5	1	1	0.41%	137078.7	5.35			
$81-3	tr|F1LYA4	1	1	0.41%	136732.2	5.35			
	R15076_4_K.FM*EDR.	713.2923	-0.00047	2	1	20.04	0		
$82-1	tr|D4ADZ9	1	1	1.97%	74639.86	5.76			
	R15076_4_K.SLFPGLI	1506.78	0.00438	3	1	24.42	0		
$83-1	tr|E9PSL7	1	1	0.34%	235307.8	6.16			
$83-2	tr|Q9QX19	1	1	0.43%	185923.9	6.22			
	R15076_4_K.VLDNQII	829.4778	-0.01494	2	1	32.61	0		
$84-1	tr|F1LNS0	1	1	0.42%	184326.9	5			
$84-2	sp|P0C6C0	1	1	0.42%	184449.1	4.98			
	R15076_4_R.IIETTSI	791.4509	-0.00183	2	1	20.43	0		
$85-1	tr|F1LNS7	1	1	0.34%	332820.1	5.74			
	R15076_4_K.DSSGGGI	980.3916	0.01884	2	1	32.76	0		
$86-1	tr|F1LQG0	1	1	1.75%	70226.5	4.71			
$86-2	sp|P54256	1	1	1.75%	70212.47	4.71			
	R15076_4_K.EITQLQA	1273.7	-0.01294	2	1	23.48	0		
$87-1	tr|F1LSL1	1	1	1.89%	33541.67	5.26			
$87-2	tr|F1LUA1	1	1	0.43%	161104.1	5.6			
$87-3	sp|Q68A21	1	1	1.90%	33417.53	5.35			
	R15076_4_R.LDIQNK.	730.4094	-0.00336	2	1	20.31	0		
$88-1	tr|F1LT35	1	1	8.33%	17733.89	10.46			

$88-2	tr|B5DES1	1	1	8.33%	17694.83	10.44			
$88-3	tr|D3ZTH8	1	1	8.33%	17752.87	10.38			
$88-4	sp|P62752	1	1	8.33%	17694.83	10.44			
	R15076_4_R.LAPDYDA	1404.701	-0.01221		2		1	66.63	0
$89-1	tr|F1LTJ8	1	1	0.81%	99025.55	6.62			
$89-2	tr|D3ZJ92	1	1	0.73%	108472.4	7.18			
	R15076_4_K.EQAKQLF	872.4948	0.01089		2		1	27.56	0
$90-1	tr|F1LV13	1	1	1.45%	73721.45	8.9			
$90-2	tr|F1M3D3	1	1	1.37%	77629.01	8.8			
$90-3	sp|Q62826	1	1	1.45%	73781.5	8.9			
	R15076_4_R.INEILSN	1114.647	-0.00612		2		1	30.84	0
$91-1	tr|F1LWF5	1	1	1.04%	157331.6	5.9			
$91-2	sp|P97603	1	1	1.09%	150636.2	5.87			
$91-3	tr|F1M4H1	1	1	1.08%	151588.3	5.78			
	R15076_4_K.M*PNDQA	1474.707	0.00124		3		1	21.5	0
$92-1	tr|F1LXQ8	1	1	1.90%	88545.65	5.04			
$92-2	tr|F1M784	1	1	1.85%	91088.45	5.08			
$92-3	tr|B5DFG9	1	1	1.85%	91060.43	5.08			
	R15076_4_R.KFLEGNS	1722.921	-0.00074		2		1	23.48	0
$93-1	tr|F1M1J6	1	1	5.93%	13639.68	9.04			
$93-2	sp|P60522	1	1	5.98%	13666.68	7.81			
$93-3	sp|Q0VGK0	1	1	5.98%	14043.88	8.67			
$93-4	sp|P60517	1	1	5.98%	13917.86	8.73			
	R15076_4_R.VPVIVEK	783.4975	-0.00114		2		1	35.73	0
$94-1	tr|F1M6F6	1	1	0.87%	102757.5	6.25			
$94-2	tr|F1LZT6	1	1	0.86%	103156.9	6.38			
$94-3	tr|F1M7E9	1	1	0.83%	107481.7	6.55			
	R15076_4_K.EIISEVG	973.5313	-0.00274		2		1	36.69	0
$95-1	tr|F1M832	1	1	0.86%	59852.78	7.73			
$95-2	tr|F1LMZ3	1	1	1.19%	45178.57	8.99			
$95-3	tr|F1LP97	1	1	1.04%	51304.51	8.53			
$95-4	tr|Q63388	1	1	4.07%	13821.7	8.06			
	R15076_4_R.FALEK.S	607.345	-0.00242		2		1	26.27	0
$96-1	tr|F1M8N8	1	1	0.30%	189643.1	6.06			
$96-2	tr|Q80XZ1	1	1	0.85%	63721.12	8.89			
$96-3	tr|M0R427	1	1	1.16%	48729.43	8.42			
$96-4	sp|P13437	1	1	1.26%	41870.45	8.09			
$96-5	tr|Q68FT8	1	1	1.02%	54892.33	5.74			
$96-6	tr|M0R3W6	1	1	1.62%	35253.13	8.46			
$96-7	tr|B0BMY3	1	1	3.11%	17933.26	7.78			
$96-8	tr|F1LZS0	1	1	1.60%	35660.57	8.27			
$96-9	tr|F7ESU6	1	1	1.31%	42858.18	8.73			
$96-10	tr|G3V9U2	1	1	1.26%	41783.42	8.37			
$96-11	tr|Q5M857	1	1	1.26%	45123.96	8.98			
	R15076_4_R.MAILR.I	603.3647	-0.00075		2		1	30.34	0
$97-1	tr|F1M9D3	1	1	1.58%	42293.78	9.64			
$97-2	tr|Q9QZG4	1	1	2.70%	25099.1	9.67			
	R15076_4_R.SMALLR.	690.3967	0.00396		2		1	31.21	0
$98-1	tr|F1MAP9	1	1	0.17%	460500	5.18			
	R15076_4_K.SGETSVF	735.3632	0.00411		2		1	24.04	0
$99-1	tr|F7FBM8	1	1	3.60%	56827.95	7			
$99-2	tr|Q63143	1	1	54.55%	3584.1	8.14			
$99-3	tr|F1LSA2	1	1	3.67%	55999.91	6.64			
$99-4	sp|P04167	1	1	3.67%	55931.92	6.8			

	R15076_4_K.ESGIAKI	2038.043	-0.01557	4	1	22.02	0		
$100-1	tr|G3V811	1	1	1.50%	83053.16	5.41			
	R15076_4_K.TLNTEGV	1203.669	0.00213	2	1	20.91	0		
$101-1	tr|G3V8J5	1	1	0.78%	109527.6	6.51			
$101-2	sp|Q9ESQ7	1	1	1.23%	70817.15	7.31			
	R15076_4_K.LVAGEYI	892.5138	-0.00072	2	1	23.39	0		
$102-1	tr|G3V931	1	1	0.91%	74245.38	6.31			
	R15076_4_M.SLPPEK.	670.377	0.00015	2	1	21.66	0		
$103-1	tr|G3V9E7	1	1	2.24%	44200.49	8.17			
$103-2	tr|G3V8E3	1	1	2.25%	43877.14	7.5			
	R15076_4_K.QREAEAT	1003.517	0.01136	2	1	34.98	0		
$104-1	tr|G3V9Q3	1	1	3.79%	49198.9	5.89			
$104-2	tr|D3ZYW2	1	1	3.96%	47074.63	5.93			
$104-3	sp|Q8VHV7	1	1	3.79%	49187.79	5.7			
$104-4	sp|Q794E4	1	1	4.10%	45729.37	5.31			
$104-5	tr|F1LV61	1	1	4.17%	44752.19	5.56			
	R15076_4_R.ATENDIY	1996.976	0.00358	2	1	69.28	0		
$105-1	tr|M0R4F6	1	1	1.15%	66914.46	8.13			
	R15076_4_R.ILHLYQK	914.5458	-0.00161	3	1	21.13	0		
$106-1	tr|M0R4Q5	1	1	1.23%	54475.59	6.28			
$106-2	tr|Q5U218	1	1	1.28%	51932.79	6.24			
	R15076_4_R.RLASPR.	699.426	0.01328	2	1	32.87	0		
$107-1	tr|M0RBQ5	1	1	6.35%	13907.94	10.31			
$107-2	tr|D3ZNH4	1	1	5.80%	15492.84	10.25			
$107-3	tr|G3V9C7	1	1	6.35%	13889.97	10.31			
$107-4	tr|D3ZLY9	1	1	6.35%	13920	10.31			
$107-5	tr|D3ZNZ9	1	1	6.35%	13994.04	10.37			
$107-6	tr|M0R4L7	1	1	6.35%	13909.96	10.31			
$107-7	tr|D4A817	1	1	6.35%	13907.99	10.31			
$107-8	tr|G3V8B3	1	1	6.35%	13905.97	10.31			
$107-9	sp|Q00715	1	1	6.40%	13990.05	10.37			
$107-10	tr|D3ZWM5	1	1	6.35%	13964.05	10.31			
$107-11	sp|Q00729	1	1	6.30%	14224.4	10.29			
	R15076_4_K.HAVSEGT	828.421	0.00012	2	1	41.91	0		
$108-1	tr|M0RBW6	1	1	4.63%	12364.1	9.4			
$108-2	tr|M0RC73	1	1	0.58%	101348.6	8.43			
	R15076_4_K.IFVDK.I	621.3606	0.00791	2	1	20.91	0		
$109-1	tr|M0RC53	1	1	2.21%	34798.11	9.26			
	R15076_4_R.KDESTGK	764.3785	0.01014	2	1	22.55	0		
$110-1	tr|M0RCX3	1	1	0.51%	258557.7	8.76			
$110-2	tr|M0R3L1	1	1	0.46%	283411.5	8.85			
$110-3	tr|F1M4F5	1	1	1.15%	115019.5	6.34			
$110-4	tr|F1M4F3	1	1	0.51%	257977	8.74			
	R15076_4_R.RKPRESI	1505.807	0.02886	4	1	24.27	0		
$111-1	sp|O08722	1	1	0.53%	103519.6	5.84			
$111-2	tr|F1LM73	1	1	0.54%	103134.7	5.68			
$111-3	tr|F1LNE2	1	1	0.53%	103672.8	5.84			
$111-4	tr|F1LRD2	1	1	0.57%	96175.54	6.34			
$111-5	tr|F1LW30	1	1	0.52%	106806.5	5.71			
$111-6	sp|Q5PPN7	1	1	1.22%	45812.78	8.22			
$111-7	sp|O08721	1	1	0.56%	98839.82	6.48			
$111-8	sp|Q761X5	1	1	0.54%	103133.7	5.72			
$111-9	tr|R9PXV5	1	1	0.55%	103719.6	8.45			
$111-10	sp|Q6DGF5	1	1	1.87%	31028.82	6.75			

$111-11	sp|P3356{	1	1	0.54%	105023.9	7.97			
	R15076_4_R.IAYLR.A	635.3875	-0.00523		2	1		29.86	0
$112-1	tr|O5515{	1	1	0.70%	96357.74	7.82			
$112-2	tr|F1LQB{	1	1	0.45%	150351.3	4.46			
$112-3	tr|G3V6A{	1	1	0.99%	65358.82	9.73			
$112-4	tr|O3578{	1	1	0.45%	151044.9	4.44			
	R15076_4_K.LQELVK.	729.4505	-0.00165		2	1		20.23	0
$113-1	sp|O88181	1	1	4.17%	41462.85	8.6			
	R15076_4_R.TPPAPHI	1804.034	0.01587		4	1		20.2	0
$114-1	tr|O88311	1	1	2.42%	36205.35	6.48			
$114-2	tr|Q7M03{	1	1	100.00%	928.13	8.75			
$114-3	tr|Q9Z0U{	1	1	2.68%	33814.89	9.39			
$114-4	tr|Q9QX8(	1	1	2.81%	30852.95	7.69			
$114-5	tr|Q9QX81	1	1	2.41%	36232.38	6.48			
	R15076_4_R.GFGFILI	928.5291	-0.00361		2	1		33.12	0
$115-1	sp|P0793(	1	1	4.42%	23603.06	4.61			
	R15076_4_K.EDPEAD(	1140.444	-0.00876		2	1		37.99	0
$116-1	sp|P1492{	1	1	0.72%	108673.9	5.78			
$116-2	tr|F1LPH{	1	1	0.72%	108744.9	5.78			
$116-3	tr|D4A1N{	1	1	0.80%	96895.09	5.92			
	R15076_4_R.SPLSVFI	777.4505	-0.00115		2	1		20.74	0
$117-1	sp|P2931{	1	1	4.64%	22591.14	10.66			
$117-2	tr|D3ZV5(	1	1	4.64%	22647.23	10.32			
	R15076_4_R.LFEGNAI	1032.584	-0.01182		3	1		20.41	0
$118-1	sp|P4212{	1	1	5.99%	36611.95	5.7			
	R15076_4_K.ITVVGV(	1973.092	-0.00049		2	1		76.03	0
$119-1	sp|P4794{	1	1	1.75%	62276.84	5.95			
	R15076_4_K.SAAEVIA	1015.553	0.00033		2	1		31.47	0
$120-1	sp|P5921{	1	1	4.24%	40068.11	5.34			
$120-2	tr|D4ABT(	1	1	4.24%	40020	5.69			
	R15076_4_R.IGAADY(	1689.844	-0.0085		2	1		40.3	0
$121-1	sp|P5938{	1	1	5.66%	24135.9	10.05			
	R15076_4_R.QRRYHAA	1425.844	-0.01448		4	1		21.34	0
$122-1	sp|P6315{	1	1	1.59%	83414.67	6.53			
	R15076_4_R.ISKQEA(	1320.716	-0.00125		2	1		21.35	0
$123-1	sp|P68101	1	1	3.81%	36107.75	5.02			
	R15076_4_K.VVTDTDI	1348.659	0.00635		2	1		60.13	0
$124-1	sp|Q0517{	1	1	6.36%	21790.01	4.5			
	R15076_4_K.ETPAASI	1316.633	-0.00129		2	1		58.93	0
$125-1	sp|Q0568{	1	1	1.37%	65401.59	6.45			
	R15076_4_R.VLEDNEI	1003.469	-0.00367		2	1		25.06	0
$126-1	tr|Q2KP1(	1	1	5.05%	21783.36	7.45			
	R15076_4_R.KMSIPES	1177.577	-0.01241		2	1		21.89	0
$127-1	sp|Q32Q9(	1	1	2.59%	22768.64	10.05			
$127-2	tr|D4A3V{	1	1	3.85%	15223.58	10.15			
	R15076_4_K.DFLSK.I	609.3243	-0.00098		2	1		24.18	0
$128-1	sp|Q499N{	1	1	2.94%	40405.49	9.17			
	R15076_4_R.LGGAGLV	1068.664	0.01027		2	1		28.39	0
$129-1	sp|Q4KLH{	1	1	3.07%	58758.93	6.26			
	R15076_4_R.LFVGNLI	1837.922	-0.0109		2	1		49.47	0
$130-1	sp|Q4V7D(	1	1	4.26%	41518.28	6.57			
$130-2	tr|Q4V8D{	1	1	4.78%	37399.9	9.07			
	R15076_4_K.ADLILSY	1898.036	0.03021		3	1		23.04	0
$131-1	tr|Q4V8H{	1	1	2.80%	53943.49	6.09			

	R15076_4_K.LEALIQF	1487.869	0.02219	2	1	21.21	0
$132-1	sp|Q5EB59	1	1	0.59%	156230	7		
	R15076_4_K.LAGRGLI	827.5461	0.0134	2	1	40.92	0
$133-1	sp|Q5FWY5	1	1	1.82%	37598.49	6.08		
	R15076_4_R.VIQEGR.	701.3941	0.00133	2	1	27.08	0
$134-1	tr|Q5M852	1	1	1.55%	43309.89	6.15		
	R15076_4_K.LPQTPK.	683.4086	-0.01196	2	1	23.29	0
$135-1	sp|Q5M9I6	1	1	3.08%	29299.34	9.86		
$135-2	tr|B1WC47	1	1	3.08%	29299.34	9.86		
	R15076_4_R.KQPTGLS	858.5043	0.01161	2	1	22.65	0
$136-1	sp|Q5PQL3	1	1	1.04%	63735.93	6.84		
$136-2	tr|B1H292	1	1	0.87%	76414.71	5.92		
	R15076_4_K.LPMVLK.	700.4426	0.00986	2	1	28.15	0
$137-1	sp|Q5U2V4	1	1	2.73%	63026.7	8.73		
	R15076_4_K.HIM*RYN	1943.943	0.00503	4	1	21.69	0
$138-1	sp|Q5XIK6	1	1	1.25%	52997.23	5.35		
	R15076_4_R.LAIEPR.	698.4195	-0.00088	2	1	23.19	0
$139-1	sp|Q5YLM1	1	1	0.48%	115300.1	6		
	R15076_4_K.DLVPR.I	599.3511	-0.00159	2	1	24.02	0
$140-1	sp|Q63092	1	1	2.58%	54104.88	5.37		
$140-2	tr|F1LR8C	1	1	2.58%	54143.98	5.37		
	R15076_4_K.GEEATGC	1422.591	-0.00134	2	1	67.57	0
$141-1	sp|Q63396	1	1	8.66%	14441.31	9.6		
	R15076_4_K.EQISDII	1260.607	-0.0061	2	1	43.74	0
$142-1	sp|Q63624	1	1	0.56%	133854.1	9.38		
	R15076_4_R.WGGLDLF	816.4363	0.0136	2	1	25.68	0
$143-1	sp|Q64620	1	1	3.93%	35158.85	5.43		
	R15076_4_R.AVPDSEF	1335.738	-0.00026	4	1	26.21	0
$144-1	tr|Q6QI30	1	1	3.80%	17670.72	9.38		
	R15076_4_-.MMKKSK.	752.4157	-0.00397	2	1	23.98	0
$145-1	sp|Q6WRH9	1	1	0.27%	284739.2	9.34		
$145-2	tr|G3V7S1	1	1	0.27%	284479.9	9.34		
	R15076_4_R.AEMRAEF	862.4199	0.00998	2	1	25.14	0
$146-1	tr|Q71DI1	1	1	10.00%	11283.74	6.09		
	R15076_4_K.DAVEDLE	1161.563	-0.00936	2	1	29.12	0
$147-1	tr|Q76MJ6	1	1	4.66%	35801.58	9.82		
$147-2	tr|F1LPL7	1	1	4.66%	35676.48	9.77		
$147-3	tr|Q3ZAV2	1	1	4.60%	36200.04	9.87		
$147-4	sp|P62961	1	1	4.66%	35729.52	9.87		
$147-5	tr|D3ZEVC	1	1	4.66%	35716.52	9.87		
	R15076_4_K.NEGSESA	1587.699	0.00033	2	1	88.15	0
$148-1	sp|Q78P75	1	1	12.36%	10349.73	6.81		
$148-2	sp|P63170	1	1	12.36%	10365.77	6.89		
	R15076_4_R.NFGSYVT	1282.606	-0.00648	2	1	51.93	0
$149-1	tr|Q7TMA9	1	1	0.59%	116460.2	9.21		
$149-2	tr|F1M9H4	1	1	0.28%	225601.4	6.39		
	R15076_4_R.LLISPR.	698.4559	-0.00075	2	1	20.87	0
$150-1	sp|Q8CGZ2	1	1	1.63%	70677.78	6.15		
	R15076_4_K.QILLENA	1170.673	-0.01635	2	1	23.81	0
$151-1	sp|Q920A6	1	1	1.77%	51174.15	5.37		
	R15076_4_K.LKWPQLS	1027.605	0.01198	2	1	22.07	0
$152-1	sp|Q9EPH2	1	1	6.53%	19846.89	4.66		
	R15076_4_K.AAATPES	1327.649	0.00118	2	1	66.31	0
$153-1	tr|Q9ER32	1	1	1.74%	72535.36	7.94		

$153-2	tr|Q9ER33	1	1	1.64%	76419.54	6.89			
$153-3	tr|Q9QWN7	1	1	1.80%	70399.01	7.59			
$153-4	tr|F1LR88	1	1	1.80%	70079.6	7.11			
	R15076_4_	R.TGFLEQ(	1250.656	0.02345	2	1	22.79	0	
$154-1	tr|Q9ESV7	1	1	5.16%	33127.74	9.65			
	R15076_4_	R.KAELAAT	1713.986	-0.00701	2	1	21.39	0	
$155-1	sp|Q9JKF8	1	1	2.14%	31713.23	9.14			
	R15076_4_	K.LAAMIK.	646.3956	0.00289	2	1	23.25	0	
$156-1	sp|Q9JMD2	1	1	1.04%	97504.7	6.22			
	R15076_4_	R.KIAVVQF	1011.62	-0.00576	3	1	20.89	0	
$157-1	sp|Q9QX69	1	1	3.01%	45239.44	7.57			
	R15076_4_	K.SLAENYF	1359.618	-0.0152	2	1	56.77	0	
$158-1	sp|Q9QZM6	1	1	0.85%	129979.6	4.59			
$158-2	tr|D3ZTI0	1	1	0.87%	126924.5	4.62			
$158-3	tr|D3ZTS6	1	1	0.92%	119735.7	4.63			
$158-4	tr|F1LN54	1	1	0.85%	130006.8	4.63			
$158-5	tr|D3ZIL4	1	1	0.94%	117955.5	4.58			
	R15076_4_	K.LIRM*GT	1275.695	0.02227	2	1	23.54	0	
$159-1	sp|Q9QZR8	1	1	0.80%	293886.5	8.44			
$159-2	tr|F1M785	1	1	0.84%	276424.7	8.5			
	R15076_4_	R.RSLSSCS	2429.128	-0.00992	4	1	20.07	0	

----- summary -----

Total prc: 322

Total prc: 159

UniPepCou	ProteinGr	Percent	ProteinCo	Percent
1	124	77.99%	260	80.75%
2	12	7.55%	27	8.39%
3	6	3.77%	10	3.11%
4	3	1.89%	5	1.55%
5	5	3.14%	8	2.48%
6	2	1.26%	3	0.93%
7	1	0.63%	2	0.62%
8	1	0.63%	1	0.31%
10	1	0.63%	2	0.62%
11	1	0.63%	1	0.31%
13	2	1.26%	2	0.62%
18	1	0.63%	1	0.31%

ExpectVal	Ions	Reference	DIFF_MOD	PI	MissClea	Modificat	MatchedTl	NumProtea
7.42E-03	0|0	Q5XIF0		3.9	0		0	2
8.13E-05	0|0	Q5XIF0		3.9	0		0	2
1.85E-01	0|0	Q5XIF0		3.9	0		0	2
5.20E-05	0|0	Q5XIF0		3.9	0		0	2
5.16E-10	0|0	Q5XIF0		3.9	0		0	2
4.76E-06	0|0	Q5XIF0		3.9	0		0	2
6.91E-06	0|0	Q5XIF0		5.32	0		0	2
7.75E-04	0|0	Q5XIF0		5.32	0		0	2
3.77E-07	0|0	Q5XIF0		5.32	0		0	2
6.97E-15	0|0	Q5XIF0		5.32	0		0	2
4.15E-09	0|0	Q5XIF0		5.32	0		0	2
2.48E-08	0|0	Q5XIF0		5.32	0		0	2
6.70E-15	0|0	Q5XIF0		5.32	0		0	2
2.08E-12	0|0	Q5XIF0		5.32	0		0	2
1.20E-07	0|0	Q5XIF0		5.32	0		0	2
1.52E-13	0|0	Q5XIF0		5.32	0		0	2
2.96E-09	0|0	Q5XIF0		5.32	0		0	2
3.01E-10	0|0	Q5XIF0		5.32	0		0	2
1.79E-15	0|0	Q5XIF0		5.32	0		0	2
7.26E-09	0|0	Q5XIF0		5.32	0		0	2
8.92E-08	0|0	Q5XIF0		5.32	0		0	2
1.07E-08	0|0	Q5XIF0		5.32	0		0	2
9.86E-14	0|0	Q5XIF0		5.32	0		0	2
1.51E-04	0|0	Q5XIF0		5.32	0		0	2
1.85E-06	0|0	Q5XIF0		5.32	0		0	2
7.95E-09	0|0	Q5XIF0		5.32	0		0	2
4.69E-08	0|0	Q5XIF0		5.32	0		0	2
3.49E-10	0|0	Q5XIF0		5.32	0		0	2
3.32E-06	0|0	Q5XIF0		5.32	0		0	2
3.02E-06	0|0	Q5XIF0		5.32	0		0	2
1.57E-07	0|0	Q5XIF0		5.32	0		0	2
3.20E-09	0|0	Q5XIF0		5.32	0		0	2
1.20E-07	0|0	Q5XIF0		5.32	0		0	2
8.96E-06	0|0	Q5XIF0		5.32	0		0	2
1.56E-07	0|0	Q5XIF0		5.32	0		0	2
4.92E-08	0|0	Q5XIF0		5.32	0		0	2
1.08E-05	0|0	Q5XIF0		5.32	0		0	2
1.23E-08	0|0	Q5XIF0		5.32	0		0	2
9.54E-02	0|0	Q5XIF0		5.32	0		0	2
7.09E-09	0|0	Q5XIF0		5.32	0		0	2
9.35E-03	0|0	Q5XIF0		8.26	0		0	2
9.02E-04	0|0	D3ZGW2 ! Q5XIF0		9.75	0		0	2
4.35E-03	0|0	Q5XIF0		3.93	0		0	2
9.55E-05	0|0	Q5XIF0		3.93	0		0	2
3.11E-04	0|0	Q5XIF0		4.48	0		0	2
1.25E-01	0|0	Q5XIF0		4.48	0		0	2
4.48E-08	0|0	Q5XIF0		4.48	0		0	2
2.39E-01	0|0	Q5XIF0		4.48	0		0	2
8.89E-09	0|0	Q5XIF0		4.48	0		0	2
7.32E-04	0|0	Q5XIF0		4.48	0		0	2
1.92E-10	0|0	Q5XIF0		4.48	0		0	2

9.70E-07	0|0	Q5XIF0	4.48	0	0	2
2.65E-06	0|0	Q5XIF0	4.48	0	0	2
8.34E-11	0|0	Q5XIF0	4.48	0	0	2
3.64E-04	0|0	Q5XIF0	4.48	0	0	2
1.52E-01	0|0	Q5XIF0	4.48	0	0	2
3.10E-09	0|0	Q5XIF0	4.48	0	0	2
8.20E-06	0|0	Q5XIF0	4.48	0	0	2
1.67E-10	0|0	Q5XIF0	4.48	0	0	2
1.59E-06	0|0	Q5XIF0	4.48	0	0	2
3.81E-12	0|0	Q5XIF0	4.48	0	0	2
6.53E-03	0|0	Q5XIF0	4.48	0	0	2
1.46E-04	0|0	Q5XIF0	4.48	0	0	2
5.50E-09	0|0	Q5XIF0	4.48	0	0	2
1.68E-04	0|0	Q5XIF0	4.48	0	0	2
3.11E-05	0|0	Q5XIF0	4.48	0	0	2
1.77E-04	0|0	Q5XIF0	4.48	0	0	2
3.11E-02	0|0	Q5XIF0	4.48	0	0	2
3.23E-06	0|0	Q5XIF0	4.48	0	0	2
4.45E-06	0|0	Q5XIF0	4.48	0	0	2
4.61E-03	0|0	Q5XIF0	4.48	0	0	2
7.73E-06	0|0	Q5XIF0	4.48	0	0	2
5.00E-07	0|0	Q5XIF0	4.48	0	0	2
3.65E-07	0|0	Q5XIF0	4.48	0	0	2
3.32E-06	0|0	Q5XIF0	4.48	0	0	2
1.18E-04	0|0	Q5XIF0	4.48	0	0	2
2.47E-02	0|0	Q5XIF0	4.53	0	0	2
8.93E-02	0|0	Q5XIF0	4.33	1	0	2
1.87E-04	0|0	Q5XIF0	6.1	0	0	2
3.67E-04	0|0	Q5XIF0	6.1	0	0	2
1.76E-03	0|0	Q5XIF0	6.1	0	0	2
1.62E-04	0|0	Q5XIF0	6.1	0	0	2
4.38E-04	0|0	Q5XIF0	6.1	0	0	2
1.48E-02	0|0	Q5XIF0	6.1	0	0	2
5.05E-02	0|0	Q5XIF0	6.1	0	0	2
3.07E-03	0|0	Q5XIF0	6.1	0	0	2
3.58E-03	0|0	Q5XIF0	9.51	1	0	2
2.66E-04	0|0	Q5XIF0	5.32	0 15.994919	0	2
9.50E-05	0|0	Q5XIF0	5.32	0 15.994919	0	2
7.56E-04	0|0	Q5XIF0	5.32	0 15.994919	0	2
3.09E-02	0|0	Q5XIF0	5.32	0 15.994919	0	2
4.00E-03	0|0	Q5XIF0	5.32	0 15.994919	0	2
1.09E-04	0|0	Q5XIF0	5.32	0 15.994919	0	2
2.19E-04	0|0	Q5XIF0	5.32	0 15.994919	0	2
4.45E-02	0|0	Q5XIF0	5.32	0 15.994919	0	2
8.33E-02	0|0	Q5XIF0	5.32	0 15.994919	0	2
4.18E-12	0|0	Q5XIF0	5.32	0	0	2
3.85E-06	0|0	Q5XIF0	5.32	0	0	2
4.33E-10	0|0	Q5XIF0	5.32	0	0	2
1.68E-13	0|0	Q5XIF0	5.32	0	0	2
3.94E-06	0|0	Q5XIF0	5.32	0	0	2
7.24E-11	0|0	Q5XIF0	5.32	0	0	2
1.54E-06	0|0	Q5XIF0	5.32	0	0	2
4.32E-09	0|0	Q5XIF0	5.32	0	0	2
1.93E-07	0|0	Q5XIF0	5.32	0	0	2

1.56E-09	0|0	Q5XIF0	5.32	0	0	2
3.32E-06	0|0	Q5XIF0	5.32	0	0	2
7.52E-08	0|0	Q5XIF0	5.32	0	0	2
7.58E-07	0|0	Q5XIF0	5.32	0	0	2
1.43E-07	0|0	Q5XIF0	5.32	0	0	2
2.31E-05	0|0	Q5XIF0	5.32	0	0	2
3.58E-08	0|0	Q5XIF0	5.32	0	0	2
1.84E-07	0|0	Q5XIF0	5.32	0	0	2
5.94E-07	0|0	Q5XIF0	5.32	0	0	2
1.77E-07	0|0	Q5XIF0	5.32	0	0	2
6.17E-07	0|0	Q5XIF0	5.32	0	0	2
1.15E-08	0|0	Q5XIF0	5.32	0	0	2
1.10E-07	0|0	Q5XIF0	5.32	0	0	2
7.65E-11	0|0	Q5XIF0	5.32	0	0	2
1.66E-06	0|0	Q5XIF0	5.32	0	0	2
4.88E-10	0|0	Q5XIF0	5.32	0	0	2
1.71E-06	0|0	Q5XIF0	5.32	0	0	2
2.14E-11	0|0	Q5XIF0	5.32	0	0	2
1.82E-07	0|0	Q5XIF0	5.32	0	0	2
5.26E-05	0|0	Q5XIF0	5.32	0	0	2
1.78E-07	0|0	Q5XIF0	5.32	0	0	2
1.45E-05	0|0	Q5XIF0	5.32	0	0	2
1.72E-06	0|0	Q5XIF0	5.32	0	0	2
1.79E-06	0|0	Q5XIF0	5.32	0	0	2
7.78E-08	0|0	Q5XIF0	5.32	0	0	2
2.71E-05	0|0	Q5XIF0	5.32	0	0	2
8.58E-06	0|0	Q5XIF0	5.32	0	0	2
3.73E-09	0|0	Q5XIF0	5.32	0	0	2
1.10E-09	0|0	Q5XIF0	5.32	0	0	2
2.98E-03	0|0	Q5XIF0	8.59	0	0	2
5.29E-02	0|0	Q5XIF0	8.59	0	0	2
1.83E-03	0|0	Q5XIF0	8.59	0	0	2
1.72E-02	0|0	Q5XIF0	8.59	0	0	2
1.79E-02	0|0	Q5XIF0	8.59	0	0	2
5.40E-03	0|0	Q5XIF0	8.59	0	0	2
5.50E-03	0|0	Q5XIF0	8.59	0	0	2
2.98E-03	0|0	Q5XIF0	8.59	0	0	2
3.22E-02	0|0	Q5XIF0	8.59	0	0	2
2.85E-03	0|0	Q5XIF0	8.59	0	0	2
1.81E-02	0|0	Q5XIF0	8.59	0	0	2
5.52E-03	0|0	Q5XIF0	8.59	0	0	2
5.50E-02	0|0	Q5XIF0	8.59	0	0	2
1.09E-01	0|0	Q5XIF0	8.59	0	0	2
4.32E-02	0|0	Q5XIF0	8.59	0	0	2
1.98E-03	0|0	Q5XIF0	8.59	0	0	2
3.26E-03	0|0	Q5XIF0	8.59	0	0	2
3.28E-03	0|0	Q5XIF0	8.59	0	0	2
2.50E-01	0|0	Q5XIF0	8.59	0	0	2
3.05E-03	0|0	Q5XIF0	8.59	0	0	2
4.14E-02	0|0	Q5XIF0	8.59	0	0	2
1.56E-01	0|0	Q5XIF0	8.59	0	0	2
4.11E-02	0|0	Q5XIF0	8.59	0	0	2
4.85E-02	0|0	Q5XIF0	8.59	0	0	2
3.07E-02	0|0	Q5XIF0	8.59	0	0	2

6.30E-05	0|0	Q5XIF0	4.37	0	0	2
4.17E-02	0|0	Q5XIF0	4.37	0	0	2
6.23E-05	0|0	Q5XIF0	4.37	0	0	2
1.66E-02	0|0	Q5XIF0	4.37	0	0	2
6.16E-06	0|0	Q5XIF0	4.37	0	0	2
4.45E-05	0|0	Q5XIF0	4.37	0	0	2
7.48E-06	0|0	Q5XIF0	4.37	0	0	2
8.82E-06	0|0	Q5XIF0	4.37	0	0	2
7.90E-04	0|0	Q5XIF0	4.37	0	0	2
2.78E-01	0|0	Q5XIF0	4.37	0	0	2
4.71E-03	0|0	Q5XIF0	4.37	0	0	2
3.06E-03	0|0	Q5XIF0	4.37	0	0	2
2.37E-02	0|0	Q5XIF0	4.37	0	0	2
2.65E-03	0|0	Q5XIF0	4.37	0	0	2
3.51E-06	0|0	Q5XIF0	4.37	0	0	2
3.90E-04	0|0	Q5XIF0	4.37	0	0	2
1.83E-03	0|0	Q5XIF0	4.37	0	0	2
1.63E-03	0|0	Q5XIF0	4.37	0	0	2
1.22E-01	0|0	Q5XIF0	4.37	0	0	2
2.10E-01	0|0	Q5XIF0	4.37	0	0	2
1.07E-01	0|0	Q5XIF0	4.37	0	0	2
3.90E-03	0|0	Q5XIF0	4.37	0	0	2
3.50E-03	0|0	Q5XIF0	4.37	0	0	2
8.05E-03	0|0	Q5XIF0	4.37	0	0	2
2.53E-03	0|0	Q5XIF0	4.37	0	0	2
2.07E-03	0|0	Q5XIF0	4.41	1	0	2
6.64E-05	0|0	Q5XIF0	4.41	1	0	2
3.92E-02	0|0	Q5XIF0	8.6	1	0	2
1.57E-02	0|0	Q5XIF0	8.6	1	0	2
4.45E-03	0|0	Q5XIF0	8.6	1	0	2
4.21E-03	0|0	Q5XIF0	8.6	1	0	2
1.03E-04	0|0	Q5XIF0	8.6	1	0	2
7.98E-03	0|0	Q5XIF0	8.6	1	0	2
8.83E-03	0|0	Q5XIF0	8.6	1	0	2
4.31E-04	0|0	Q5XIF0	8.6	1	0	2
5.87E-03	0|0	Q5XIF0	8.6	1	0	2
1.57E-01	0|0	Q5XIF0	8.6	1	0	2
3.32E-04	0|0	Q5XIF0	8.6	1	0	2
2.80E-03	0|0	Q5XIF0	8.6	1	0	2
9.50E-03	0|0	Q5XIF0	8.6	1	0	2
2.06E-03	0|0	Q5XIF0	8.6	1	0	2
9.48E-04	0|0	Q5XIF0	8.6	1	0	2
1.73E-03	0|0	Q5XIF0	8.6	1	0	2
3.11E-13	0|0	Q5XIF0	3.99	0 15.994919	0	2
2.13E-02	0|0	Q5XIF0	3.99	0 15.994919	0	2
1.56E-02	0|0	Q5XIF0	3.99	0 15.994919	0	2
8.32E-06	0|0	Q5XIF0	3.99	0	0	2
6.71E-14	0|0	Q5XIF0	3.99	0	0	2
1.14E-11	0|0	Q5XIF0	3.99	0	0	2
5.19E-12	0|0	Q5XIF0	3.99	0	0	2
6.11E-12	0|0	Q5XIF0	3.99	0	0	2
1.17E-07	0|0	Q5XIF0	3.99	0	0	2
1.19E-05	0|0	Q5XIF0	3.99	0	0	2
4.59E-12	0|0	Q5XIF0	3.99	0	0	2

4.80E-05	0|0	Q5XIF0	3.99	0	0	2
6.56E-03	0|0	Q5XIF0	3.99	0	0	2
9.70E-13	0|0	Q5XIF0	3.99	0	0	2
1.13E-08	0|0	Q5XIF0	3.99	0	0	2
1.85E-12	0|0	Q5XIF0	3.99	0	0	2
2.06E-06	0|0	Q5XIF0	3.99	0	0	2
4.06E-10	0|0	Q5XIF0	3.99	0	0	2
2.16E-04	0|0	Q5XIF0	3.99	0	0	2
3.62E-14	0|0	Q5XIF0	3.99	0	0	2
2.04E-10	0|0	Q5XIF0	3.99	0	0	2
4.61E-12	0|0	Q5XIF0	3.99	0	0	2
2.33E-12	0|0	Q5XIF0	3.99	0	0	2
1.30E-11	0|0	Q5XIF0	3.99	0	0	2
1.57E-02	0|0	Q5XIF0	4.37	0	0	2
1.39E-03	0|0	Q5XIF0	4.37	0	0	2
2.33E-05	0|0	Q5XIF0	4.37	0	0	2
4.26E-07	0|0	Q5XIF0	4.37	0	0	2
4.50E-05	0|0	Q5XIF0	4.37	0	0	2
9.74E-05	0|0	Q5XIF0	4.37	0	0	2
1.43E-04	0|0	Q5XIF0	4.37	0	0	2
6.96E-04	0|0	Q5XIF0	4.37	0	0	2
2.74E-04	0|0	Q5XIF0	4.37	0	0	2
4.48E-05	0|0	Q5XIF0	4.37	0	0	2
5.48E-04	0|0	Q5XIF0	4.37	0	0	2
3.48E-04	0|0	Q5XIF0	4.37	0	0	2
2.26E-04	0|0	Q5XIF0	4.37	0	0	2
1.37E-04	0|0	Q5XIF0	4.37	0	0	2
3.01E-04	0|0	Q5XIF0	4.37	0	0	2
5.31E-04	0|0	Q5XIF0	4.37	0	0	2
7.36E-05	0|0	Q5XIF0	4.37	0	0	2
5.63E-04	0|0	Q5XIF0	4.37	0	0	2
6.52E-03	0|0	Q5XIF0	4.37	0	0	2
1.68E-05	0|0	Q5XIF0	4.37	0	0	2
7.01E-04	0|0	Q5XIF0	4.37	0	0	2
5.66E-05	0|0	Q5XIF0	4.37	0	0	2
9.28E-06	0|0	Q5XIF0	4.37	0	0	2
2.02E-02	0|0	Q5XIF0	4.37	0	0	2
2.70E-03	0|0	Q5XIF0	4.37	0	0	2
5.05E-03	0|0	Q5XIF0	4.37	0	0	2
2.36E-03	0|0	Q5XIF0	4.37	0	0	2
1.92E-02	0|0	Q5XIF0	6.71	0	0	2
2.64E-04	0|0	Q5XIF0	6.71	0	0	2
2.43E-03	0|0	Q5XIF0	6.71	0	0	2
3.30E-04	0|0	Q5XIF0	6.71	0	0	2
3.57E-03	0|0	Q5XIF0	6.71	0	0	2
1.87E-03	0|0	Q5XIF0	6.71	0	0	2
5.94E-03	0|0	Q5XIF0	6.71	0	0	2
3.01E-04	0|0	Q5XIF0	6.71	0	0	2
1.69E-02	0|0	Q5XIF0	6.71	0	0	2
8.09E-03	0|0	Q5XIF0	6.71	0	0	2
4.23E-02	0|0	Q5XIF0	6.71	0	0	2
6.00E-04	0|0	Q5XIF0	6.71	0	0	2
6.81E-03	0|0	Q5XIF0	6.71	0	0	2
2.55E-02	0|0	Q5XIF0	6.71	0	0	2

9.28E-04	0|0	Q5XIF0	6.71	0		0	2
1.51E-02	0|0	Q5XIF0	6.71	0		0	2
6.34E-04	0|0	Q5XIF0	6.71	0		0	2
3.52E-03	0|0	Q5XIF0	6.71	0		0	2
2.68E-04	0|0	Q5XIF0	6.71	0		0	2
3.46E-02	0|0	Q5XIF0	6.71	0		0	2
1.66E-03	0|0	Q5XIF0	6.71	0		0	2
1.26E-03	0|0	Q5XIF0	6.71	0		0	2
2.61E-02	0|0	Q5XIF0	6.71	0		0	2
5.48E-04	0|0	Q5XIF0	6.71	0		0	2
6.70E-04	0|0	Q5XIF0	6.71	0		0	2
4.67E-04	0|0	Q5XIF0	6.71	0		0	2
2.24E-03	0|0	Q5XIF0	6.71	0		0	2
1.44E-01	0|0	Q5XIF0	6.71	0		0	2
3.16E-04	0|0	Q5XIF0	6.71	0		0	2
9.87E-04	0|0	Q5XIF0	6.71	0		0	2
9.69E-04	0|0	Q5XIF0	6.71	0		0	2
2.21E-03	0|0	Q5XIF0	6.71	0		0	2
6.60E-04	0|0	Q5XIF0	6.71	0		0	2

1.44E-02	0|0	P85108/Q3KRE8/Q6P9	4.14	0	15.994919	0	2
1.09E-03	0|0	P85108/Q3KRE8/Q6P9	4.14	0		0	2
1.28E-02	0|0	P85108/Q3KRE8/Q6P9	4.68	1		0	2
6.17E-02	0|0	P85108/Q3KRE8/Q4QR	9.75	0	15.994919	0	2
8.16E-05	0|0	P85108/Q3KRE8/Q4QR	9.75	0		0	2
1.14E-01	0|0	P85108/Q3KRE8/P698	6.73	0		0	2
3.31E-04	0|0	P85108/Q3KRE8/Q4QR	5.83	0	15.994919	0	2
2.30E-04	0|0	P85108/Q3KRE8/Q4QR	5.83	0		0	2
4.37E-04	0|0	P85108/Q3KRE8/Q6P9	6	0		0	2
1.95E-02	0|0	P85108/Q3KRE8/Q4QR	4.03	0	15.994919	0	2
5.50E-04	0|0	P69897	4.37	0		0	2
3.71E-04	0|0	P85108/Q3KRE8/Q4QR	5.84	0		0	2
2.44E-04	0|0	P85108/Q3KRE8/Q4QR	5.84	0		0	2
5.14E-03	0|0	P85108/Q3KRE8/Q4QR	5.84	0		0	2
1.28E-06	0|0	Q4QRB4/Q6P9T8/P698	8.75	0		0	2
6.45E-03	0|0	P85108/Q3KRE8/Q4QR	6	0	15.994919	0	2
7.05E-06	0|0	P69897	6	0		0	2
4.73E-01	0|0	P85108/Q3KRE8/Q6P9	8.46	0		0	2

1.44E-02	0|0	P85108/Q3KRE8/Q6P9	4.14	0	15.994919	0	2
1.09E-03	0|0	P85108/Q3KRE8/Q6P9	4.14	0		0	2
1.28E-02	0|0	P85108/Q3KRE8/Q6P9	4.68	1		0	2
6.17E-02	0|0	P85108/Q3KRE8/Q4QR	9.75	0	15.994919	0	2
8.16E-05	0|0	P85108/Q3KRE8/Q4QR	9.75	0		0	2
1.14E-01	0|0	P85108/Q3KRE8/P698	6.73	0		0	2
2.43E-05	0|0	P85108/Q3KRE8/Q6P9	5.75	0		0	2
3.31E-04	0|0	P85108/Q3KRE8/Q4QR	5.83	0	15.994919	0	2
2.30E-04	0|0	P85108/Q3KRE8/Q4QR	5.83	0		0	2
4.37E-04	0|0	P85108/Q3KRE8/Q6P9	6	0		0	2
1.95E-02	0|0	P85108/Q3KRE8/Q4QR	4.03	0	15.994919	0	2
3.71E-04	0|0	P85108/Q3KRE8/Q4QR	5.84	0		0	2
2.44E-04	0|0	P85108/Q3KRE8/Q4QR	5.84	0		0	2
5.14E-03	0|0	P85108/Q3KRE8/Q4QR	5.84	0		0	2
1.69E-04	0|0	Q3KRE8	6	0		0	2

6.45E-03	0|0	P85108/Q3KRE8/Q4QI	6	0 15.99491	0	2
4.73E-01	0|0	P85108/Q3KRE8/Q6P9	8.46	0	0	2
7.18E-04	0|0	P85108/Q3KRE8	8.75	0	0	2
1.44E-02	0|0	P85108/Q3KRE8/Q6P9	4.14	0 15.99491	0	2
1.09E-03	0|0	P85108/Q3KRE8/Q6P9	4.14	0	0	2
1.28E-02	0|0	P85108/Q3KRE8/Q6P9	4.68	1	0	2
6.17E-02	0|0	P85108/Q3KRE8/Q4QI	9.75	0 15.99491	0	2
8.16E-05	0|0	P85108/Q3KRE8/Q4QI	9.75	0	0	2
1.14E-01	0|0	P85108/Q3KRE8/P698	6.73	0	0	2
2.43E-05	0|0	P85108/Q3KRE8/Q6P9	5.75	0	0	2
3.31E-04	0|0	P85108/Q3KRE8/Q4QI	5.83	0 15.99491	0	2
2.30E-04	0|0	P85108/Q3KRE8/Q4QI	5.83	0	0	2
4.37E-04	0|0	P85108/Q3KRE8/Q6P9	6	0	0	2
3.71E-04	0|0	P85108/Q3KRE8/Q4QI	5.84	0	0	2
2.44E-04	0|0	P85108/Q3KRE8/Q4QI	5.84	0	0	2
5.14E-03	0|0	P85108/Q3KRE8/Q4QI	5.84	0	0	2
1.28E-06	0|0	Q4QRB4/Q6P9T8/P698	8.75	0	0	2
6.45E-03	0|0	P85108/Q3KRE8/Q4QI	6	0 15.99491	0	2
4.73E-01	0|0	P85108/Q3KRE8/Q6P9	8.46	0	0	2
1.94E-04	0|0	P68370/Q6P9V9/Q6AY	5.84	0	0	2
5.87E-05	0|0	P68370/Q6P9V9/Q6AY	4.03	0	0	2
3.63E-04	0|0	P68370/Q6P9V9/Q6AY	4.03	0	0	2
2.84E-02	0|0	P68370/Q6P9V9/Q6AY	5.84	0	0	2
3.75E-04	0|0	P68370/Q6P9V9/Q6AY	4.03	0	0	2
7.28E-03	0|0	P68370/Q6P9V9/Q68I	5.81	0	0	2
3.34E-08	0|0	P68370/Q6P9V9/Q6AY	3.92	0	0	2
1.55E-02	0|0	P68370/Q6P9V9/Q6AY	4.03	0	0	2
5.69E-04	0|0	P68370/Q6P9V9/Q6AY	5.84	0	0	2
1.39E-02	0|0	P68370/Q6P9V9/Q6AY	6.07	0	0	2
2.65E-05	0|0	P68370/Q6P9V9/Q6AY	6.07	0	0	2
2.30E-06	0|0	P68370/Q6P9V9/Q6AY	6.75	0	0	2
2.35E-03	0|0	P68370/Q6P9V9/Q6AY	6.75	0	0	2
6.17E-02	0|0	P85108/Q3KRE8/Q4QI	9.75	0 15.99491	0	2
8.16E-05	0|0	P85108/Q3KRE8/Q4QI	9.75	0	0	2
3.31E-04	0|0	P85108/Q3KRE8/Q4QI	5.83	0 15.99491	0	2
2.30E-04	0|0	P85108/Q3KRE8/Q4QI	5.83	0	0	2
4.37E-04	0|0	P85108/Q3KRE8/Q6P9	6	0	0	2
1.95E-02	0|0	P85108/Q3KRE8/Q4QI	4.03	0 15.99491	0	2
3.71E-04	0|0	P85108/Q3KRE8/Q4QI	5.84	0	0	2
2.44E-04	0|0	P85108/Q3KRE8/Q4QI	5.84	0	0	2
5.14E-03	0|0	P85108/Q3KRE8/Q4QI	5.84	0	0	2
1.28E-06	0|0	Q4QRB4/Q6P9T8/P698	8.75	0	0	2
6.45E-03	0|0	P85108/Q3KRE8/Q4QI	6	0 15.99491	0	2
2.37E-05	0|0	Q4QRB4	6.75	0	0	2
1.23E-04	0|0	Q4QRB4	6.75	0	0	2
7.94E-03	0|0	F1LQW3/Q4KM71	4.03	0	0	2
3.56E-04	0|0	F1LQW3/Q4KM71	9.8	0	0	2
7.29E-02	0|0	F1LQW3/Q4KM71	4.37	0	0	2

4.09E-05	0|0	Q5FVM4/F1LQW3/Q4KM	4.21	0	0	2
2.46E-03	0|0	Q5FVM4/F1LQW3/Q4KM	4.21	0	0	2
1.15E-03	0|0	F1LQW3/Q4KM71	9.76	0	0	2
2.53E-04	0|0	F1LQW3/Q4KM71	9.76	0	0	2
2.46E-06	0|0	F1LQW3/Q4KM71	3.92	0	0	2
1.06E-01	0|0	F1LQW3/Q4KM71/O547	4.75	0	0	2
2.84E-02	0|0	P68370/Q6P9V9/Q6A	5.84	0	0	2
7.28E-03	0|0	P68370/Q6P9V9/Q68	5.81	0	0	2
1.55E-02	0|0	P68370/Q6P9V9/Q6A	4.03	0	0	2
4.23E-01	0|0	Q6AY56	5.38	1	0	2
1.39E-02	0|0	P68370/Q6P9V9/Q6A	6.07	0	0	2
2.65E-05	0|0	P68370/Q6P9V9/Q6A	6.07	0	0	2
2.30E-06	0|0	P68370/Q6P9V9/Q6A	6.75	0	0	2
2.35E-03	0|0	P68370/Q6P9V9/Q6A	6.75	0	0	2
7.14E-04	0|0	Q8K4V4/M0RBS9/G3V	5.84	0	0	2
5.36E-02	0|0	Q8K4V4/M0RBS9	5.72	0	0	2
5.81E-02	0|0	Q8K4V4/M0RBS9/G3V	6.1	0	0	2
6.74E-02	0|0	Q8K4V4/M0RBS9/G3V	4.25	0	0	2
1.85E-03	0|0	Q8K4V4/M0RBS9	6	0	0	2
2.53E-07	0|0	Q8K4V4/M0RBS9	5.4	0	0	2
8.13E-03	0|0	Q5FVM4	4.37	0	0	2
4.09E-05	0|0	Q5FVM4/F1LQW3/Q4KM	4.21	0	0	2
2.46E-03	0|0	Q5FVM4/F1LQW3/Q4KM	4.21	0	0	2
3.54E-05	0|0	Q5FVM4	8.26	0	0	2
5.70E-04	0|0	Q5FVM4	8.26	0	0	2
6.72E-04	0|0	Q5FVM4	4.53	0	0	2
2.18E-06	0|0	Q5FVM4	4.75	0	0	2
3.12E-05	0|0	P62630/M0R757/F1M	4.68	0	0	2
7.43E-02	0|0	P62630/P62632/M0R	8.75	0	0	2
6.46E-04	0|0	P62630/P62632/M0R	8.33	0	0	2
5.38E-03	0|0	P62630/M0R757/M0R	6.74	0	0	2
1.57E-04	0|0	P62630/M0R757/M0R	6.74	0	0	2
5.07E-05	0|0	P62630/M0R757/F1M	8.75	0	0	2
2.20E-02	0|0	Q6IG01/Q6IG00/Q6P	5.84	0	0	2
3.53E-05	0|0	P47819/Q6IG03/Q6I	4.37	0	0	2
2.81E-04	0|0	Q6IG02/Q6IG00/Q6I	4.25	0	0	2
1.26E-02	0|0	Q6IG00/Q6IG05/Q6I	6.14	1	0	2
7.27E-06	0|0	Q6P6Q2/Q4FZU2/Q6I	4.03	0	0	2
2.20E-04	0|0	P48721/F1M953	3.84	0	0	2
1.11E-04	0|0	P48721/M0R972/F1M	4.53	0	0	2
1.30E-05	0|0	P48721/F1M953	5.81	0	0	2
1.19E-03	0|0	P48721/F1M953	4.37	0	0	2
4.46E-03	0|0	P48721/M0R972/F1M	4.37	0	0	2

3.19E-02	0|0	P63018/M0R8M9/M0RI	5.84	0	0	2
2.16E-02	0|0	P14659/P63018/F1LZ	5.84	0	0	2
5.26E-03	0|0	P63018/D3ZH98/M0RI	5.84	0	0	2
2.00E-04	0|0	P14659/P63018/M0R8	4.14	0	0	2
2.92E-08	0|0	P06761 ! P55063 !	4.37	0	0	2
2.20E-02	0|0	Q6IG01/Q6IG00/Q6P6	5.84	0	0	2
7.80E-04	0|0	Q6IG01/Q6P6Q2/Q107	6.18	2	0	2
3.92E-04	0|0	Q6IG01/Q6P6Q2/Q107	6.18	2	0	2
1.26E-02	0|0	Q6IG00/Q6IG05/Q6IF	6.14	1	0	2
7.27E-06	0|0	Q6P6Q2/Q4FZU2/Q6IC	4.03	0	0	2
2.49E-05	0|0	P47727/M0R3X6/Q9QZ	8.07	0	0	2
1.43E-04	0|0	P47727/M0R3X6/A0AC	6	0	0	2
1.79E-02	0|0	P47727/B2GV72/M0R3	5.21	0	0	2
8.83E-02	0|0	P47727/B2GV72/M0R3	5.21	0	0	2
2.52E-07	0|0	P47727/M0R3X6	4.09	0	0	2
3.25E-06	0|0	P15205/F1LRL9/B0BN	4.37	0	0	2
8.52E-02	0|0	P15205/F1LRL9	5.66	0	0	2
5.35E-03	0|0	P34926/P15205/G3V7	4.37	0	0	2
1.20E-02	0|0	P15205/F1LRL9/B0BN	6.74	0	0	2
8.97E-02	0|0	P97576	4.8	0	0	2
7.21E-03	0|0	P97576	4.8	0	0	2
2.73E-05	0|0	P97576	4.37	0	0	2
1.99E-06	0|0	P97576	11	0	0	2
1.72E-03	0|0	P01946/B1H216	5.16	0	0	2
3.81E-04	0|0	P01946/B1H216	4.75	0	0	2
1.64E-03	0|0	P01946/B1H216	8.5	0	0	2
1.39E-02	0|0	P02091/P11517/Q626	5.32	0	0	2
1.48E-04	0|0	P02091/P11517	5.81	0	0	2
9.15E-08	0|0	P11517	4.37	0	0	2
2.94E-03	0|0	Q63716	4.21	0	0	2
1.56E-01	0|0	Q63716	8.76	0	0	2
5.83E-02	0|0	Q63716	4.37	0	0	2
6.97E-04	0|0	Q9JJ54	4.14	0	0	2
8.01E-02	0|0	Q9JJ54	5.84	0	0	2
5.84E-02	0|0	Q3SWU3/M0RAX7/M0R9	8.75	0	0	2
3.88E-02	0|0	P60711/P63259/D3ZF	4.21	0	0	2
7.31E-06	0|0	P62738/P60711/P68C	4.14	0	0	2
1.34E-04	0|0	P62738/P60711/P68C	8.76	0	0	2

1.80E-01	0|0	D3ZH98	4.03	0	0	2
6.84E-02	0|0	D3ZH98	4.03	0	0	2
5.26E-03	0|0	P63018/D3ZH98/M0RI	5.84	0	0	2
2.08E-03	0|0	D3ZN95	5.4	0	0	2
2.02E-04	0|0	D3ZN95	6	0	0	2
1.28E-01	0|0	D3ZPW4	4.37	0	0	2
6.84E-02	0|0	O88453/M0RBF0 ! D4	5.81	0	0	2
5.92E-03	0|0	P04256/Q5I0M7/Q6P6	6.85	0	0	2
3.10E-03	0|0	P04256/Q5I0M7/Q6P6	4.13	0	0	2
1.83E-02	0|0	P00762	5.51	2	0	2
4.58E-08	0|0	P00762	4.4	0	0	2
4.58E-02	0|0	P04905/P08010/G3V9	5.83	0	0	2
6.24E-05	0|0	P04905/G3V983/Q6LI	4.21	0	0	2
2.92E-08	0|0	P06761 ! P55063 !	4.37	0	0	2
2.33E-04	0|0	P06761	6.07	1	0	2
3.86E-04	0|0	P35704	8.31	0	0	2
2.78E-03	0|0	P35704	5.84	0	0	2
3.76E-06	0|0	P04797/M0R590/D3ZC	4	0	0	2
6.94E-04	0|0	P04797/D3ZEN2/M0R6	8.75	0	0	2
3.33E-04	0|0	P15146/Q78DZ1/F1LN	5.84	0	0	2
2.96E-02	0|0	P15146/F1LNK0/F1MA	4.53	0	0	2
6.24E-04	0|0	Q6AYI1/B6DTP5	4.53	0	0	2
7.20E-04	0|0	Q6AYI1/B6DTP5	4.37	0	0	2
2.68E-02	0|0	Q9JLT0/G3V9Y1	4.57	1	0	2
1.73E-01	0|0	Q9JLT0/Q63862/Q628	4.68	1	0	2
1.67E-02	0|0	D4A8X8	4.37	0	0	2
6.04E-03	0|0	D4A8X8	4.37	0	0	2

9.82E-03	0|0	D4A8X8	4.37	0	0	2
1.74E-02	0|0	D4A8X8	4.37	0	0	2
3.83E-03	0|0	D4A8X8	4.37	0	0	2
8.22E-03	0|0	D4A8X8	4.37	0	0	2
1.01E-02	0|0	D4A8X8	4.37	0	0	2
2.75E-02	0|0	D4A8X8	4.37	0	0	2
8.32E-03	0|0	D4A8X8	4.37	0	0	2
1.50E-01	0|0	D4A8X8	4.37	0	0	2
8.77E-02	0|0	D4A4U8	8.69	1	0	2
2.05E-01	0|0	D4A4U8	8.69	1	0	2
1.01E-01	0|0	D4A4U8	8.69	1	0	2
2.91E-01	0|0	M0RA91	8.75	1	0	2
4.80E-01	0|0	M0RA91	8.75	1	0	2
3.06E-01	0|0	M0RA91	8.75	1	0	2
2.12E-01	0|0	Q4KLM0	6	0	0	2
4.47E-01	0|0	Q4KLM0	6	0	0	2
4.37E-01	0|0	Q4KLM0	6	0	0	2
2.74E-01	0|0	D3ZB99	6	0	0	2
3.59E-01	0|0	D3ZB99	6	0	0	2
6.84E-02	0|0	D4A7T9	5.84	2	0	2
1.18E-01	0|0	D4A7T9	5.84	2	0	2
1.95E-01	0|0	Q8VIP2/C7C5T1	5.88	0	0	2
1.65E-01	0|0	Q8VIP2/C7C5T1	5.88	0	0	2
2.09E-01	0|0	A0JN13	4.56	1	0	2
3.26E-01	0|0	A1A5R0/F1LPJ8	8.59	0	0	2
2.84E-01	0|0	A1L1J7	4.37	0	0	2
2.52E-01	0|0	A5I9F0/Q9WUL3/F1LF	6	0	0	2
1.69E-01	0|0	B0BN75	4.79	1	0	2
6.93E-02	0|0	B0BNK9	11	1	0	2
1.42E-01	0|0	B0BNM8	8.59	1 15.994919	0	2
2.72E-02	0|0	Q5U2W6 ! B1WBT0	4.37	0 15.994919	0	2
9.91E-01	0|0	B1WBX7/G3V8Q1	4.03	0	0	2

3.92E-02	0|0	B2GUW2	9.47	0	0	2
8.22E-02	0|0	P04906/B6DYQ7/D4A8	5.59	0	0	2
1.26E-02	0|0	G3V6F8/C0LP03	6	0	0	2
6.64E-02	0|0	P81795/C9WPN6/W8CI	4.53	0	0	2
3.35E-02	0|0	D3Z8B0	9.72	0	0	2
1.66E-01	0|0	D3Z954	12	1 15.99491	0	2
1.31E-02	0|0	D3ZE49/M0R780	5.84	0	0	2
1.18E-01	0|0	D3ZFN9	10.84	2	0	2
1.02E-02	0|0	D3ZFQ7	11	1	0	2
3.94E-01	0|0	D4A2P1/D3ZG47	9.99	2	0	2
7.13E-02	0|0	Q6MG97/A0A0A0MY44	8.22	0	0	2
3.36E-01	0|0	D3ZH42	8.72	0	0	2
2.48E-02	0|0	D3ZHF9/A0A096MK16	11	1	0	2
1.89E-01	0|0	D3ZI44	6.24	1	0	2
7.02E-01	0|0	D3ZNQ2	8.75	1 15.99491	0	2

3.83E-01	0|0	D3ZPP5	4.53	0		0	2
1.77E-01	0|0	A0JPL2/D3ZRE3	8.8	0		0	2
2.99E-01	0|0	D3ZXS4	6.92	1		0	2
5.19E-01	0|0	D3ZYM5	5.81	0		0	2
9.87E-02	0|0	D4A0G9	6.1	0		0	2
3.57E-01	0|0	D4A1X2/D4A2C3/M0R8	6.1	0		0	2
1.14E-02	0|0	D4A3X1	8.26	0		0	2
2.65E-01	0|0	D4A5W5	12	1		0	2
2.07E-01	0|0	D4A6C6	9.72	0		0	2
1.90E-01	0|0	D4A7X9	9.2	2		0	2
3.36E-02	0|0	D4A961	9.75	0		0	2
5.87E-02	0|0	D4A9Q5	6.86	1		0	2
5.26E-02	0|0	A2VD13/D4AA10	5.84	0		0	2
1.47E-02	0|0	F1LZH7/D4AAW3	5.84	0		0	2
3.32E-02	0|0	Q9JKB5/F1LYA4 ! D4	4.37	0	15.99491	0	2
1.15E-01	0|0	D4ADZ9	4.78	1		0	2
3.59E-02	0|0	Q9QX19/E9PSL7	5.81	0		0	2
6.36E-01	0|0	P0C6C0/F1LNS0	6	0		0	2
5.30E-04	0|0	F1LNS7	4.03	0		0	2
2.61E-01	0|0	P54256/F1LQG0	4.53	0		0	2
5.83E-01	0|0	Q68A21/F1LSL1 ! F1	5.84	0		0	2

1.24E-05	0|0	P62752/B5DES1/D3ZT	3.93	0		0	2
5.79E-02	0|0	D3ZJ92 ! F1LTJ8	8.85	1		0	2
2.64E-02	0|0	Q62826/F1M3D3/F1LV	6	0		0	2
4.56E-01	0|0	P97603/F1M4H1/F1LW	8.5	1	15.994919	0	2
1.30E-01	0|0	F1LXQ8/F1M784/B5DF	8.59	2		0	2
2.26E-03	0|0	P60517/Q0VGK0 ! P6	5.97	0		0	2
8.26E-03	0|0	F1M6F6/F1LZT6/F1M7	4.53	0		0	2
4.34E-02	0|0	F1M832/F1LMZ3/F1LF	6	0		0	2
2.67E-02	0|0	F1M8N8 ! Q80XZ1/MC	9.5	0		0	2
2.84E-02	0|0	F1M9D3/Q9QZG4	9.47	0		0	2
1.30E-01	0|0	F1MAP9	5.72	0		0	2

5.63E-01	0|0	P04167/F1LSA2/F7FE	8.29	1	0	2
2.83E-01	0|0	G3V811	8.41	1	0	2
9.96E-02	0|0	Q9ESQ7/G3V8J5	6	0	0	2
1.02E-01	0|0	G3V931	5.72	0	0	2
9.53E-03	0|0	G3V8E3/G3V9E7	6.14	1	0	2
4.81E-06	0|0	Q8VHV7/G3V9Q3/D3ZY	4.37	0	0	2
2.32E-01	0|0	M0R4F6	8.6	0	0	2
2.41E-02	0|0	M0R4Q5/Q5U218	12	1	0	2
8.99E-04	0|0	Q00729/Q00715/D3ZW	6.75	0	0	2
6.88E-01	0|0	M0RC73 ! M0RBW6	5.84	0	0	2
1.33E-01	0|0	M0RC53	6.07	1	0	2
3.26E-01	0|0	F1M4F3/M0R3L1/M0RC	8.59	2	0	2

3.27E-02	0|0	Q5PPN7 ! O08721/Q7	8.75	0	0	2
3.03E-01	0|0	G3V6A2 ! O55157/F1	6	0	0	2
5.16E-01	0|0	O88181	12	0	0	2
1.98E-02	0|0	Q9QX80/Q9QX81/O883	8.75	0	0	2
5.24E-04	0|0	P07936	3.83	0	0	2
1.80E-01	0|0	P14925/F1LPH3/D4A1	8.47	0	0	2
2.29E-01	0|0	P29314/D3ZV50	6	0	0	2
5.05E-07	0|0	P42123	8.22	0	0	2
5.42E-02	0|0	P47942	5.72	0	0	2
7.55E-03	0|0	P59215/D4ABT0	4.03	0	0	2
2.81E-01	0|0	P59382	11	2	0	2
2.37E-01	0|0	P63155	6.14	1	0	2
4.48E-05	0|0	P68101	3.92	0	0	2
2.93E-05	0|0	Q05175	4.53	0	0	2
1.50E-01	0|0	Q05683	4	0	0	2
1.95E-01	0|0	Q2KP10	4.79	1	0	2
1.01E-01	0|0	Q32Q90 ! D4A3V2	5.84	0	0	2
1.06E-02	0|0	Q499N4	12	1	0	2
8.52E-04	0|0	Q4KLH4	3.92	0	0	2
4.07E-01	0|0	Q4V7D6/Q4V8D5	8.64	0	0	2

1.52E-01	0|0	Q4V8H9	6.75	0	0	2
1.53E-03	0|0	Q5EB59	11	1	0	2
1.75E-01	0|0	Q5FWY5	5.97	0	0	2
3.05E-02	0|0	Q5M852	8.75	0	0	2
3.12E-01	0|0	Q5M9I6/B1WC47	10	1	0	2
6.34E-02	0|0	Q5PQL3 ! B1H292	8.75	0	0	2
2.40E-01	0|0	Q5U2V4	9.4	2 15.99491	0	2
4.37E-02	0|0	Q5XIK6	6	0	0	2
1.36E-01	0|0	Q5YLM1	5.84	0	0	2
9.54E-07	0|0	Q63092/F1LR80	4.25	0	0	2
1.95E-03	0|0	Q63396	3.84	0	0	2
7.75E-02	0|0	Q63624	5.84	0	0	2
1.02E-01	0|0	Q64620	6.12	1	0	2
5.46E-02	0|0	Q6QI30	10.3	2	0	2
7.73E-02	0|0	Q6WRH9/G3V7S1	6.19	1	0	2
9.56E-02	0|0	Q71DI1	3.92	0	0	2
1.55E-08	0|0	P62961/D3ZEV0/Q3Z	4.25	0	0	2
2.66E-04	0|0	P63170/Q78P75	6.75	0	0	2
6.10E-02	0|0	Q7TMA9 ! F1M9H4	9.75	0	0	2
7.72E-02	0|0	Q8CGZ2	4.53	0	0	2
2.70E-01	0|0	Q920A6	11	1	0	2
5.20E-06	0|0	Q9EPH2	4.53	0	0	2

3.00E-01	0|0	Q9ER33/Q9ER32/F1LF	5.66	0		0	2
1.10E-01	0|0	Q9ESV7	8.59	2		0	2
2.16E-01	0|0	Q9JKF8	8.75	0		0	2
6.31E-02	0|0	Q9JMD2	8.59	1		0	2
1.06E-04	0|0	Q9QX69	4.37	0		0	2
3.57E-01	0|0	Q9QZM6/D3ZTI0/F1LN	11.7	2	15.99491	0	2
1.88E-01	0|0	Q9QZR8/F1M785	6.14	1	15.99491	0	2

ıseTermini
